# An updated phylogeny and adaptive evolution within Amaranthaceae *s.l*. inferred from multiple phylogenomic datasets

**DOI:** 10.1002/ece3.70013

**Published:** 2024-07-14

**Authors:** Hao Xu, Yuqin Guo, Mingze Xia, Jingya Yu, Xiaofeng Chi, Yun Han, Xiaoping Li, Faqi Zhang

**Affiliations:** ^1^ Key Laboratory of Adaptation and Evolution of Plateau Biota, Northwest Institute of Plateau Biology and Institute of Sanjiangyuan National Park Chinese Academy of Sciences Xining China; ^2^ University of Chinese Academy of Sciences Beijing China; ^3^ Qinghai National Park Research Monitoring and Evaluation Center Xining China; ^4^ School of Pharmacy Weifang Medical University Weifang China; ^5^ Qinghai Provincial Key Laboratory of Crop Molecular Breeding Xining China

**Keywords:** adaptive evolution, Amaranthaceae *s.l.*, divergence time, incomplete lineage sorting, plastome structure

## Abstract

Amaranthaceae *s.l.* is a widely distributed family consisting of over 170 genera and 2000 species. Previous molecular phylogenetic studies have shown that Amaranthaceae *s.s.* and traditional Chenopodiaceae form a monophyletic group (Amaranthaceae *s.l.*), however, the relationships within this evolutionary branch have yet to be fully resolved. In this study, we assembled the complete plastomes and full‐length ITS of 21 Amaranthaceae *s.l.* individuals and compared them with 38 species of Amaranthaceae *s.l*. Through plastome structure and sequence alignment analysis, we identified a reverse complementary region approximately 5200 bp long in the genera *Atriplex* and *Chenopodium*. Adaptive evolution analysis revealed significant positive selection in eight genes, which likely played a driving role in the evolution of Amaranthaceae *s.l.*, as demonstrated by partitioned evolutionary analysis. Furthermore, we found that about two‐thirds of the examined species lack the *ycf*15 gene, potentially associated with natural selection pressures from their adapted habitats. The phylogenetic tree indicated that some genera (*Chenopodium*, *Halogeton*, and Subtr. Salsolinae) are paraphyletic lineages. Our results strongly support the clustering of Amaranthaceae *s.l.* with monophyletic traditional Chenopodiaceae (Clades I and II) and Amaranthaceae *s.s.* After a comprehensive analysis, we determined that cytonuclear conflict, gene selection by adapted habitats, and incomplete lineage sorting (ILS) events were the primary reasons for the inconsistent phylogeny of Amaranthaceae *s.l.* During the last glacial period, certain species within Amaranthaceae *s.l.* underwent adaptations to different environments and began to differentiate rapidly. Since then, these species may have experienced morphological and genetic changes distinct from those of other genera due to intense selection pressure.

## Graphical abstract

Our results strongly demonstrated that Amaranthaceae *s.l*. was clustered with monophyletic traditional Chenopodiaceae (Clades I and II) and Amaranthaceae *s.s*. based on multiple phylogenomic datasets. Phylogenetic analysis of adaptive evolution showed that positively selected genes played a driving role in the evolutionary direction of Amaranthaceae *s.l*.

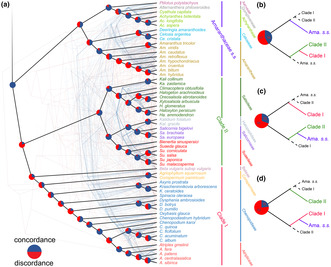

## 1 INTRODUCTION

Amaranthaceae *sensu lato*, belonging to the order Caryophyllales within the phylum Angiosperm, is considered a moderately large family that includes both the Chenopodiaceae (this name has been abolished and we now refer to it as traditional Chenopodiaceae) and the Amaranthaceae *sensu stricto* (Mabberley, 2017). Many economically important plants, such as quinoa (*Chenopodium quinoa* Willd.), spinach (*Spinacia oleracea* L.), and sugar beet (*Beta vulgaris* L.), are predominantly classified under traditional Chenopodiaceae (Alvarez‐Jubete et al., 2010; Kupper, 2005; Zhang et al., 2016). These plants provide non‐conventional protein sources due to their excellent nutritional value, significantly contributing to human nutrition (Caruso et al., 2018; Pellegrini et al., 2018). Most traditional Chenopodiaceae plants benefit from saline growth media (Akhani et al., 2007; Chen et al., 2020; Shegebayev et al., 2023; Sultanova et al., 2021). A considerable proportion of arable land worldwide is affected by salinity, increasing the demand for salt‐tolerant crops (Slama et al., 2015). Most Amaranthaceae *s.l*. thrive in arid and saline environments, with leaves and bracts adapted into fleshy forms, enabling them to grow in saline habitats and potentially produce useful crops on salty soils (Akhani et al., 2007; Piirainen et al., 2017). The high salt tolerance and bioactive compound content in traditional Chenopodiaceae plants such as *Kali* Mill. and *Salicornia* L., along with their functional and health properties, make these glassworts important candidates for future fresh and processed foods (Karakas et al., 2017; Ventura et al., 2015).

Over the past two decades, Amaranthaceae *s.s*. has gained significant recognition, with some species widely recognized as troublesome and economically harmful agronomic weeds in cropping systems (Beckie, 2006; Webster & Nichols, 2012). Additionally, numerous Amaranthaceae *s.s*. species, such as *Alternanthera philoxeroides* (Mart.) Griseb., *Al. pungens* Kunth, *Amaranthus spinosus* L., *Am. hybridus* L., and *Am. retroflexus* L., have been identified as invasive species, posing risks to ecosystems (Abdyeva et al., 2021; Lu et al., 2010; Pan, 2007; Ren et al., 2023). Recent studies have also highlighted the nutritional value of certain Amaranthaceae *s.s*. species, such as *Am. caudatus* L., which serve as potential sources of bioactive compounds (Martinez‐Lopez et al., 2020). However, these findings remain primarily theoretical.

Although Amaranthaceae *s.l*. provides abundant nutrition and protein as crops, the phylogenetic relationships between Amaranthaceae *s.s*. and traditional Chenopodiaceae remain unsatisfactorily resolved due to morphological similarities at the species‐level and ILS events at the molecular level (Bao et al., 2003; Frankton, 1968; Huang et al., 2020; Tutin, 1964). Correct classification of Amaranthaceae *s.l*. taxa is essential to utilize traditional Chenopodiaceae as raw materials for economically important plants and crops. Recent molecular systematic studies have confirmed the monophyly of both Amaranthaceae *s.s*. and Chenopodiaceae, indicating their close relationship (The Angiosperm Phylogeny Group, 2016; Yang et al., 2018; Yao et al., 2019). However, the phylogenetic position of Betoideae remains unresolved, leading to ongoing debates about Chenopodiaceae. Consequently, scholars have considered Chenopodiaceae an untenable family, resulting in its merger with Amaranthaceae *s.s*. (Brignone et al., 2021; Mabberley, 2017; The Angiosperm Phylogeny Group, 2016).

Amaranthaceae *s.l*. faces issues in species classification and phylogenetic relationships between lineages in current systematic studies. We supplemented molecular data of Amaranthaceae *s.l*. worldwide by collecting local dominant plant groups. We hypothesized that different climate conditions and habitat characteristics specific to each region have influenced the evolution of genes in Amaranthaceae *s.l*., leading to adaptive differentiation. Against this background, we studied the systematic evolution of Amaranthaceae *s.l*. using plastomes and nuclear ribosomal DNA collected from the Qaidam Basin and previously published data. Therefore, we aimed to clarify (1) the phylogenetic relationship between the two major lineages of Amaranthaceae *s.l*. (Amaranthaceae *s.s*. and traditional Chenopodiaceae); (2) to identify protein‐coding genes (PCGs) that contributed to adaptive divergence and explore the significance of these genes on the evolution of Amaranthaceae *s.l*.; and (3) to infer the diversification of Amaranthaceae *s.l*. through molecular and temporal evolution.

## 2 MATERIALS AND METHODS

### 2.1 Plant sources

We collected young and fresh leaves from 21 Amaranthaceae *s.l*. samples, comprising 20 samples from the saline‐alkali environments of the Qaidam Basin in Qinghai Province and one sample from Gaize County in the Tibetan Autonomous Region, each representing one individual species (Table 1). The plant materials were dried in silica gel and stored at −20°C. Vouchers for all the samples were deposited into the Qinghai‐Tibetan Plateau Museum of Biology (QTPMB), University of Chinese Academy of Sciences. Additionally, we included plastomes of non‐replicated species from earlier studies in this analysis (Table S1).

**TABLE 1:** Sample collection information and GenBank accession for plastomes of Amaranthaceae *s.l.*

Species	Voucher No.	Location	Longitude	Latitude	Accession No.
*Krascheninnikovia ceratoides*	QXA0006	Delingha, QH	96°08′11.51″ E	37°22′46.33″ N	ON149841
*Corispermum pamiricum*	QXA0009	Delingha, QH	96°08′11.51″ E	37°22′46.33″ N	ON149842
*Xylosalsola arbuscula*	QXA0010	Delingha, QH	96°08′11.51″ E	37°22′46.33″ N	ON149843
*Kali zaidamicum*	QXA0020	Delingha, QH	96°38′13.06″ E	37°18′38.43″ N	MZ230595
*Haloxylon ammodendron*	QXA0021	Delingha, QH	96°38′13.06″ E	37°18′38.43″ N	ON149844
*Halogeton arachnoideus*	QXA0032	Delingha, QH	96°38′13.14″ E	37°10′03.50″ N	ON149845
*Halogeton glomeratus*	QXA0156	Delingha, QH	97°21′59.97″ E	36°58′25.13″ N	ON149846
*Agriophyllum squarrosum*	QXA0171	Delingha, QH	97°34′23.39″ E	36°21′30.66″ N	ON149847
*Kalidium foliatum*	QXA0175	Delingha, QH	97°34′23.39″ E	36°21′30.66″ N	ON149848
*Chenopodiastrum hybridum*	QXA0193	Dulan, QH	98°07′14.83″ E	35°53′35.57″ N	ON149849
*Atriplex patens*	QXA0233	Dulan, QH	97°40′26.02″ E	36°02′02.18″ N	ON149850
*Krascheninnikovia arborescens*	QXA0288	Dulan, QH	98°11′30.14″ E	36°20′59.08″ N	ON149851
*Atriplex sibirica*	QXA0326	Wulan, QH	98°55′32.55″ E	36°50′38.98″ N	ON149852
*Chenopodium karoi*	QXA0358	Tianjun, QH	98°54′11.99″ E	37°12′07.69″ N	ON149853
*Axyris prostrata*	QXA0379	Tianjun, QH	98°39′46.69″ E	37°34′16.50″ N	ON149854
*Kali collinum*	QXA0430	Dulan, QH	98°40′46.68″ E	36°33′30.33″ N	ON149855
*Oreosalsola abrotanoides*	QXA160730011	Mangya, QH	91°45′55.18″ E	37°51′38.62″ N	ON149856
*Atriplex fera*	QXA160805022	Dachaidan, QH	94°31′17.59″ E	38°05′39.00″ N	ON149857
*Kalidium gracile*	QXA160806003	Delingha, QH	97°04′59.98″ E	37°20′36.21″ N	ON149858
*Climacoptera obtusifolia*	QXA160806022	Delingha, QH	96°30′45.72″ E	37°24′28.53″ N	ON149859
*Suaeda corniculata*	zhangfq866	Gaize XZ	83°19′78.22″ E	32°44′58.80″ N	ON149860

### 2.2 DNA extraction and sequencing

Total genomic DNA was extracted from Amaranthaceae *s.l*. leaves using the modified CTAB protocol of Yang et al. (2014). We determined DNA concentration using a microspectrophotometer (Nanodrop 2000 and Qubit 4, USA) and DNA quality using 1% agarose gel electrophoresis. After the library was finished and the quality controlled, sequencing was performed on an Illumina NovaSeq 6000 platform (Illumina Inc., San Diego, CA, USA) at Novogene in Tianjin, China, generating 10 Gb of high‐quality 150‐bp paired‐end reads per sample.

### 2.3 Sequence assembly and annotation

We filtered the raw sequence reads using Trimmomatic v.0.38 (Bolger et al., 2014) to remove adaptors and low‐quality sequences with the parameters: SLIDINGWINDOW:5:20, LEADING:5, TRAILING:5, MINLEN:50, and the remaining parameters were set to default. Clean reads were mapped to the seed database (i.e., embryophyte plastomes database, default: embplant_pt) using Bowtie2 (Langmead & Salzberg, 2012). Target‐associated reads were assembled into contigs with SPAdes v3.14.0 (Bankevich et al., 2012) in GetOrganelle v.1.7.5 (Jin et al., 2020). Contigs were aligned to the reference genome (GenBank accession number: NC_041200.1), and assembled plastomes were annotated using Geneious v4.8.3 (Kearse et al., 2012). De novo assembly was performed with GetOrganelle v1.7.5 to obtain nuclear ribosomal DNA, and full‐length ITS sequences were extracted using ITSx v1.1.2 (Bengtsson‐Palme et al., 2013; Table S2).

The completed plastomes were annotated using the Geseq (Tillich et al., 2017) from CHLOROBOX (https://chlorobox.mpimp‐golm.mpg.de/geseq.html) and Plastid Genome Annotator (PGA) (Qu et al., 2019). Manual examination and adjustment of start/stop codons and exon/intron boundaries were done using Geneious v4.8.3 (Kearse et al., 2012). The correction of all tRNA genes was confirmed using tRNAscan‐SE v2.0 (Chan et al., 2021; http://lowelab.ucsc.edu/tRNAscan‐SE/index.html).

### 2.4 Genome structure and comparative analysis

Plastome characteristics, including the number of genes, nucleotide polymorphism, and structural diagrams, were analyzed. We visualized the divergence of 59 complete plastomes using the mVISTA online genome comparison tool (Frazer et al., 2004). Nucleotide diversity (Pi) was calculated using 79 de‐redundant PCGs and 127 intergenic spacer regions with DnaSP v.6.12 (Rozas et al., 2017).

### 2.5 Adaptive evolution detection

To investigate positive selection in Amaranthaceae *s.l*., we analyzed the ratio (*ω* = *d*_N_/*d*_S_) of non‐synonymous (*d*_N_) and synonymous (*d*_S_) mutations using PAML v.4.9j (Yang, 2007). Maximum likelihood (ML) phylogenetic trees were reconstructed with IQ‐TREE v.2.0.3 (Nguyen et al., 2015) using a best‐fit model obtained by ModelFinder v.1.6.12 (Kalyaanamoorthy et al., 2017) with 10,000 bootstrap replicates. Site models (seqtype = 1, model = 0, NSsites = 1, 2, 7, 8) in CodeML from PAML v4.9j were used to identify positively selected sites in protein‐coding regions. Likelihood ratio tests (LRT) using the site models (including M1 [neutral] vs. M2 [positive selection] and M7 [beta] vs. M8 [beta and ω]) and the branch‐site models (including null hypothesis; Yang & Nielsen, 2002) identified positive sites with significant posterior probability support (*p* ≥ .99) and compared values of different models (Whelan & Goldman, 1999). Branch‐site models were employed to further assess genes identified as under selection in site models, testing the alternative hypothesis using the branch of traditional Chenopodiaceae (Clades I and II) as the foreground branch, with the corresponding null hypothesis (*ω* = 1) examined for likelihood ratio. Positive selection sites were identified using the BEB method (Yang et al., 2005).

### 2.6 Phylogenetic analysis

All plastomes [59 Amaranthaceae *s.l*. individuals and 5 out groups (*Cornus capitata*, *Marcgravia coriacea*, *Phaulothamnus spinescens*, *Quintinia verdonii*, and *Simmondsia chinensis*)] were extracted into 87 PCGs using Python scripts from PhyloSuite v.1.2.2 (Zhang et al., 2020). To ensure the accuracy of the phylogenetic analysis, we reconstructed the phylogeny tree of Amaranthaceae *s.l*. using five datasets: (i) PCG regions, (ii) complete plastomes of 64 species, (iii) full‐length ITS, (iv) positively selected PCGs, and (v) non‐positively selected PCGs. All datasets were aligned using default parameters in MAFFT v.7.313 (Katoh & Standley, 2013) and frameshift variations in datasets (i, iv, and v) were adjusted using MACSE v.2.03 (Ranwez et al., 2011). Poorly aligned regions were excluded using Gblocks v.0.91b (Castresana, 2000) with default parameters. The best‐fitting model for the trimmed datasets was determined using ModelFinder v.1.6.12 (Kalyaanamoorthy et al., 2017), with the parameter “Criterion” set to “Corrected Akaike information criterion (AICc).”

ML analysis was conducted with IQ‐TREE v.2.0.3 (Nguyen et al., 2015) using the GTR + F + I + G4 model, 10,000 bootstrap replicates, and a minimum correlation coefficient of 0.99. Bayesian Inference (BI) analysis was performed with MRBAYES v.3.2.6 (Ronquist & Huelsenbeck, 2003) using GTR + F + I + G4 model, with 1,000,000 generations of Markov chain Monte Carlo (MCMC) simulations, sampling every 1000 generations. The average standard deviation of split frequencies was restricted to less than 0.01, indicating convergence of the BI tree topology. The first 25% of the trees were discarded as burn‐in, and a consensus tree was obtained. Both BI and ML analyses were performed identically for all datasets.

For the coalescence‐based method, we constructed 81 single‐gene ML trees based on dataset (i) shared across all species using IQ‐TREE v.2.0.3 (st = CODON11, m = MFP) and merged these tree files to reconstruct a species tree using ASTRAL v.5.7.8 (Zhang et al., 2018). Using the 81 shared PCGs, we examined discordance between gene trees and the species tree with Phyparts v.0.0.1 (Smith et al., 2015). We used Toytree v.2.0.1 (Eaton, 2020) to build cloud tree plots to display conflicts between the single‐gene trees and the species tree.

### 2.7 Divergence time estimation

To estimate the evolutionary age of Amaranthaceae *s.l*., we employed BEAST v.2.6.6 (Bouckaert et al., 2019) to calibrate divergence time based on datasets (i), (iv), and (v). An input file was generated using BEAUti v.2.6.6 (Bouckaert et al., 2019), selecting GTR as the substitution model with a strict clock model and a birth–death model of speciation, based on jModelTest2 (Posada, 2008) results indicating the best model as GTR + F + I + G4. Three well‐dated fossils were used to calibrate relevant nodes. The stem node of Chenopodieae (Clade I) was calibrated using the Lower Miocene seed fossil of *Parvangula randeckensis* Hiltermann & Schmitz with a lognormal distribution (mean = 1.0, SD = 0.5, and offset = 23.3 Ma; Gregor, 1982; Kadereit et al., 2003). The age of the node representing the crown of Amaranthaceae *s.l*. was assigned using the pollen fossil *Polyporina cribraria* Srivastava from the Upper Cretaceous of Canada, with a lognormal distribution (mean = 1.0, SD = 0.5, and offset = 66.0 Ma; Srivastava, 1970). The crown node of Salicornieae Ulbr. was calibrated using the pollen fossil *Salicornites massalongoi* Principi with a normal distribution (mean = 29.0 and sigma = 2.5), based on loose divergence time estimates (35–23 Mya; Principi, 1926). Three independent MCMC analyses were run for 200 million generations, sampling every 5000 generations until the effective sample size (ESS) value was more than 200 for all relevant parameters in Tracer v.1.7.2 (Rambaut et al., 2018). We used LogCombiner v.2.6.6 (Bouckaert et al., 2019) to combine the three runs and obtained a time‐scaled phylogenetic tree using TreeAnnotator v.2.6.6 (Bouckaert et al., 2019) discarding the first 10% of the trees as burn‐in.

## 3 RESULTS

### 3.1 Molecular features of the plastomes

We analyzed the complete plastomes of 59 Amaranthaceae *s.l*. taxa and five outgroups, revealing a typical circular tetramerous structure comprising a large single copy (LSC), a small single copy (SSC), and two inverted repeats (IRs; Table S1). The total length of Amaranthaceae *s.l*. plastomes was similar, ranging from 149,722 to 155,108 bp, and the GC content remained stable, varying from 36.2 to 37.6%. Most species had a highly identical genetic composition in terms of gene order and number. The number of PCGs encoded by plastomes ranged from 84 to 87, with subtle variation due to the presence or absence of *ycf*15 (Table 2). The number of transfer RNA genes was mostly 37, although a few species had lost *trn*S^GCU^, *trn*G^GCC^, *trn*N^GUU^, and others due to genetic mutations. Ribosomal RNA genes were highly conserved and had not changed in number.

**TABLE 2:** Statistical analysis of missing genes for Amaranthaceae *s.l*.

Species	Missing genes	
	PCGs	tRNA
*Atriplex centralasiatica*	*ycf*15	
*Atriplex gmelinii*	*ycf*15	
*Atriplex patens*	*ycf*15	
*Atriplex fera*	*ycf*15	
*Atriplex sibirica*	*ycf*15	
*Axyris prostrata*	*ycf*15	
*Chenopodium karoi*	*ycf*15	
*Krascheninnikovia arborescens*	*ycf*15	
*Krascheninnikovia ceratoides*	*ycf*15	
*Agriophyllum squarrosum*	*ycf*15	
*Corispermum pamiricum*	*ycf*15	
*Kalidium foliatum*	*ycf*15	*trn*S‐GCU
*Kalidium gracile*	*ycf*15	
*Halogeton glomeratus*	*ycf*15	
*Haloxylon ammodendron*	*psa*I	
*Oreosalsola abrotanoides*	*ycf*15	
*Xylosalsola arbuscula*	*ycf*15	
*Kali collinum*	*ycf*15	
*Bienertia sinuspersici*	*ycf*15	
*Suaeda corniculata*	*ycf*15	
*Achyranthes aspera*	*ycf*15	
*Achyranthes bidentata*	*ycf*15	
*Achyranthes longifolia*	*ycf*15	*trn*G‐GCC
*Cyathula capitata*	*ycf*15*, pet*G	
*Ptilotus polystachyus*	*ycf*15	
*Amaranthus blitum*	*ycf*15	
*Amaranthus caudatus*	*ycf*15	
*Amaranthus hybridus*	*ycf*15	
*Amaranthus cruentus*	*ycf*15	
*Amaranthus hypochondriacus*	*ycf*15	
*Amaranthus retroflexus*	*ycf*15	
*Amaranthus tricolor*	*ycf*15*, rpo*C2	
*Amaranthus viridis*	*ycf*15	
*Celosia argentea*	*ycf*15	
*Celosia cristata*	*ycf*15	
*Deeringia amaranthoides*	*ycf*15	*trn*N‐GUU, *trn*N‐GUU‐copy2
*Alternanthera philoxeroides*	*ycf*15	*trn*E‐UUC
*Beta vulgaris* subsp. *vulgaris*	*ycf*15	*trn*G‐UCC, *trn*I‐GAU‐copy2

### 3.2 Structural and sequence divergence analysis of the plastomes

**FIGURE 1:**
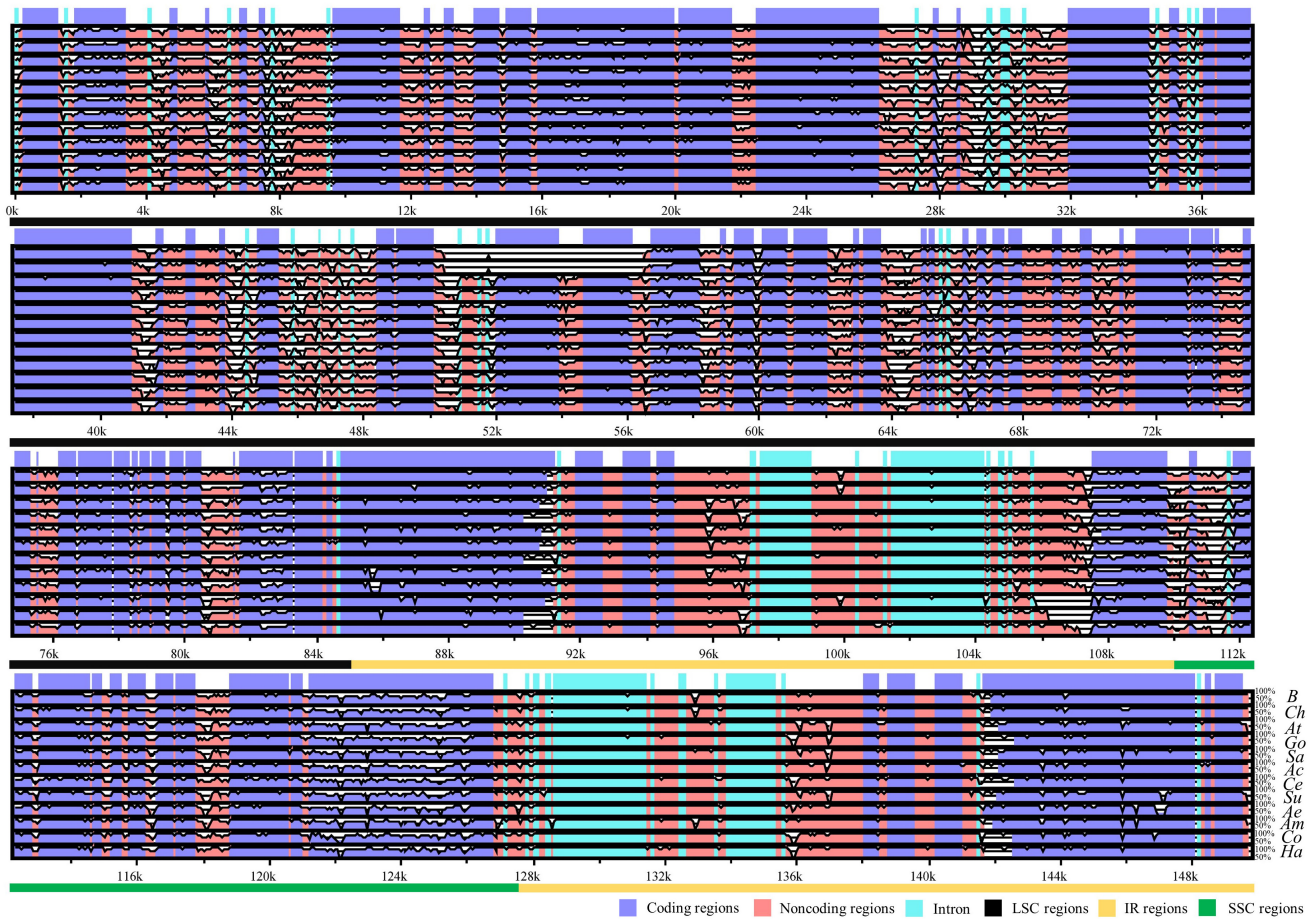
mVISTA‐based visualization of whole plastomes comparison for Amaranthaceae *s.l*. 13 species was selected to represent their tribes respectively to compare the characteristics of plastome (Be = Beteae, Ch = Chenopodieae, At = Atripliceae, Go = Gomphreneae, Sa = Salsoleae, Ac = Achyrantheae, Ce = Celosieae, Su = Suaedeae, Ae = Aeryeae, Am = Amarantheae, Co = Corispermeae, Ha = Halopeplideae) and *Beta vulgaris* subsp. *vulgaris* was used as the reference.

We used the plastomes of 13 species of Amaranthaceae *s.l*. as representatives of their tribes to compare the characteristics of plastomes among these species (Figure 1). Our finding showed that two IR regions were more conserved than the other two regions, and coding regions displayed low divergence in sequence characteristics. We also found that two tribes (Atripliceae and Chenopodieae) had about 5200 bp of the reverse complementary region in the middle and downstream of the LSC regions, involving the tandem gene sequence (*rbc*L‐*atp*B‐*atp*E‐*trn*M^CAU^‐*trn*V^UAC^; Figure S1). Nucleotide diversity (Pi value) was detected separately for PCGs and non‐coding regions (Figure S2). Pi values of PCGs ranged from 0.0074 (*ndh*B) to 0.3934 (*ycf*15), indicating large sequence diversity among different tribes in Amaranthaceae *s.l*. Among all 127 non‐coding regions, Pi values varied from 0.0033 (*ycf*2‐*trn*I^CAU^) to 0.2707 (*psb*I‐*trn*S^GCU^). Four loci (*trn*I^GAU^‐intron1‐*trn*I^GAU^‐intron2, *rps*16‐*trn*Q^UUG^, *trn*G^GCC^‐*trnf*M^CAU^, *trn*S^GGA^‐*rps*4) had the most variable Pi values (>0.2).

### 3.3 Adaptive evolution analysis

We selected 79 de‐redundant PCGs across 59 Amaranthaceae *s.l*. individuals for positive selection, excluding duplicated genes in IRs. This identified eight genes that were positively selected through statistical analysis (Table 3). These genes included hypothetical protein reading frame genes 1 and 2 (*ycf*1 and *ycf*2), *acc*D, *ccs*A, *ndh*A, *rbc*L, *rpl*22, and *rps*12. Based on M2 (positive selection) and M8 (beta and ω) site models, we identified positive selection sites within these genes: *ycf*2 contained 16 and 18 sites, *ycf*1 contained 15 and 15 sites, *acc*D contained 5 and 5 sites, *rpl*22 contained 3 and 3 sites, *rbc*L contained 3 and 7 sites, *ccs*A contained 1 and 2 sites, *ndh*A contained 1 and 3 sites, and *rps*12 contained 1 and 1 sites (Table S3). The branch‐site models with traditional Chenopodiaceae as foreground branches identified that *ycf*2 and *ycf*1 each contained 16 sites, *acc*D, *rpl*22, and *rbc*L each contained 4 sites, *ndh*A contained 2 sites, and *rps*12 contained 1 site, while *ccs*A did not contain any sites (Table S4). Both the branch‐site models and the site model exhibited highly similar results, with statistically significant positively selected sites (*p* < .01).

**TABLE 3:** Likelihood ratio tests (LRT) for eight positively selected sites based on the site models and branch‐site models (M1: Neutral; M2: Selection; M7: Beta; M8: Beta & gamma; Model A: The branch‐site models; Model null: Null hypothesis).

Gene	2ΔlnL			Chi‐squared test (*p*‐value)		
	M1 versus M2	M7 versus M8	Model A versus Model null	M1 versus M2	M7 versus M8	Model A versus Model null
*ycf*2	112.05	116.28	125.93	4.67E‐25	5.62E‐26	0
*ycf*1	107.65	125.81	115.08	4.22E‐24	4.81E‐28	0
*acc*D	33.36	41.09	31.56	5.70E‐08	1.19E‐09	1.93E‐08
*rpl*22	19.41	14.76	12.14	6.10E‐05	6.24E‐04	4.93E‐04
*rbc*L	15.46	16.71	24.05	4.40E‐04	2.35E‐04	9.37E‐07
*ccs*A	14.54	21.29	9.26	6.98E‐04	2.38E‐05	2.33E‐02
*rps*12	10.22	10.35	4.05	6.02E‐03	5.65E‐03	4.42E‐02
*ndh*A	9.76	17.68	16.41	7.59E‐03	1.44E‐04	5.09E‐05

### 3.4 Phylogenetic relationships of Amaranthaceae *s.l.*

**FIGURE 2:**
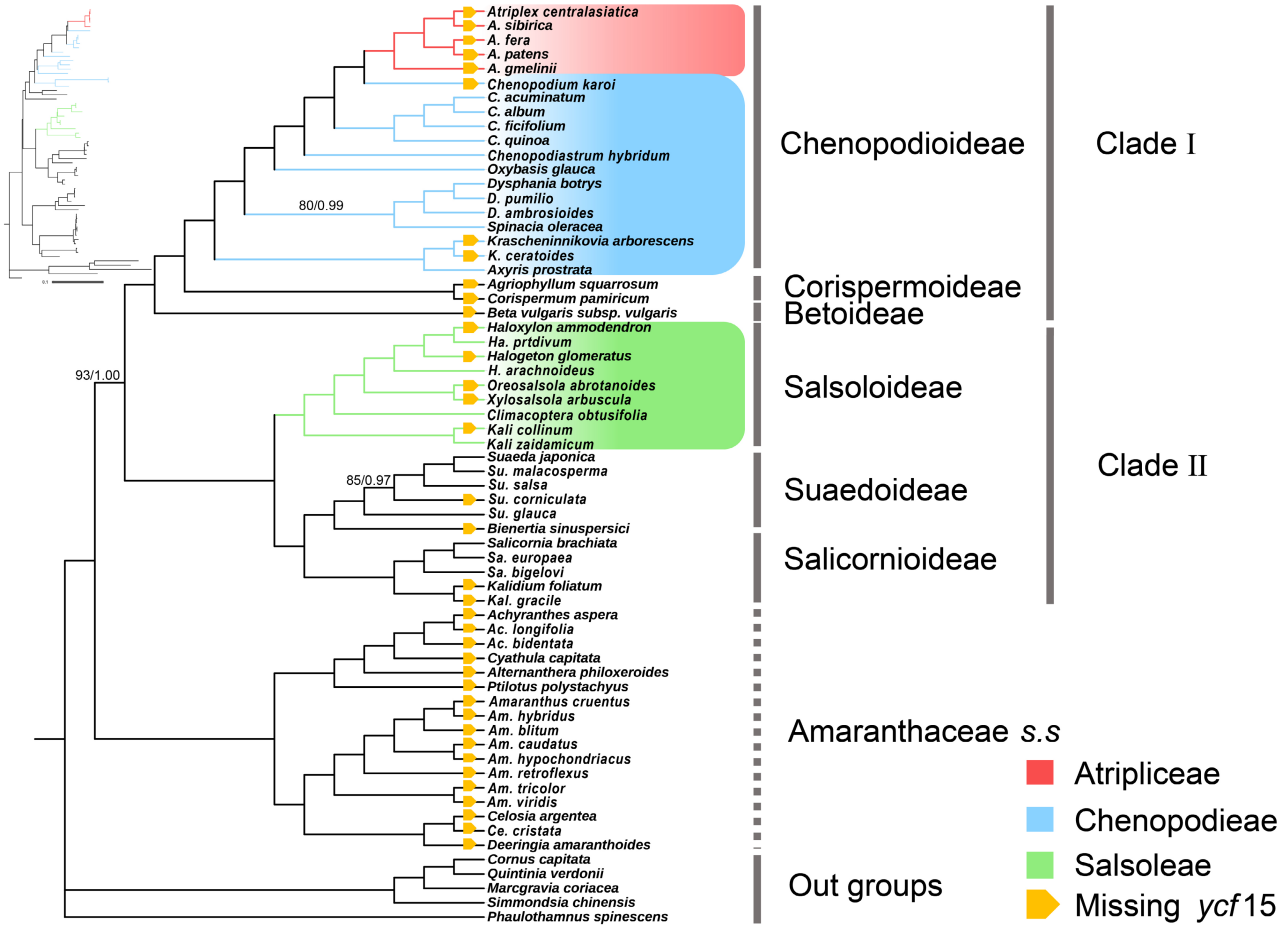
Phylogenetic tree of the family Amaranthaceae *s.l*. revealed by the concatenated datasets of 87 protein‐coding genes using Maximum Likelihood (ML) and Bayesian inference (BI). Support values above the branches are ML bootstrap values/Bayesian posterior probabilities. Nodes without digits indicate 100% bootstrap value/1.0 posterior probability.

**FIGURE 3:**
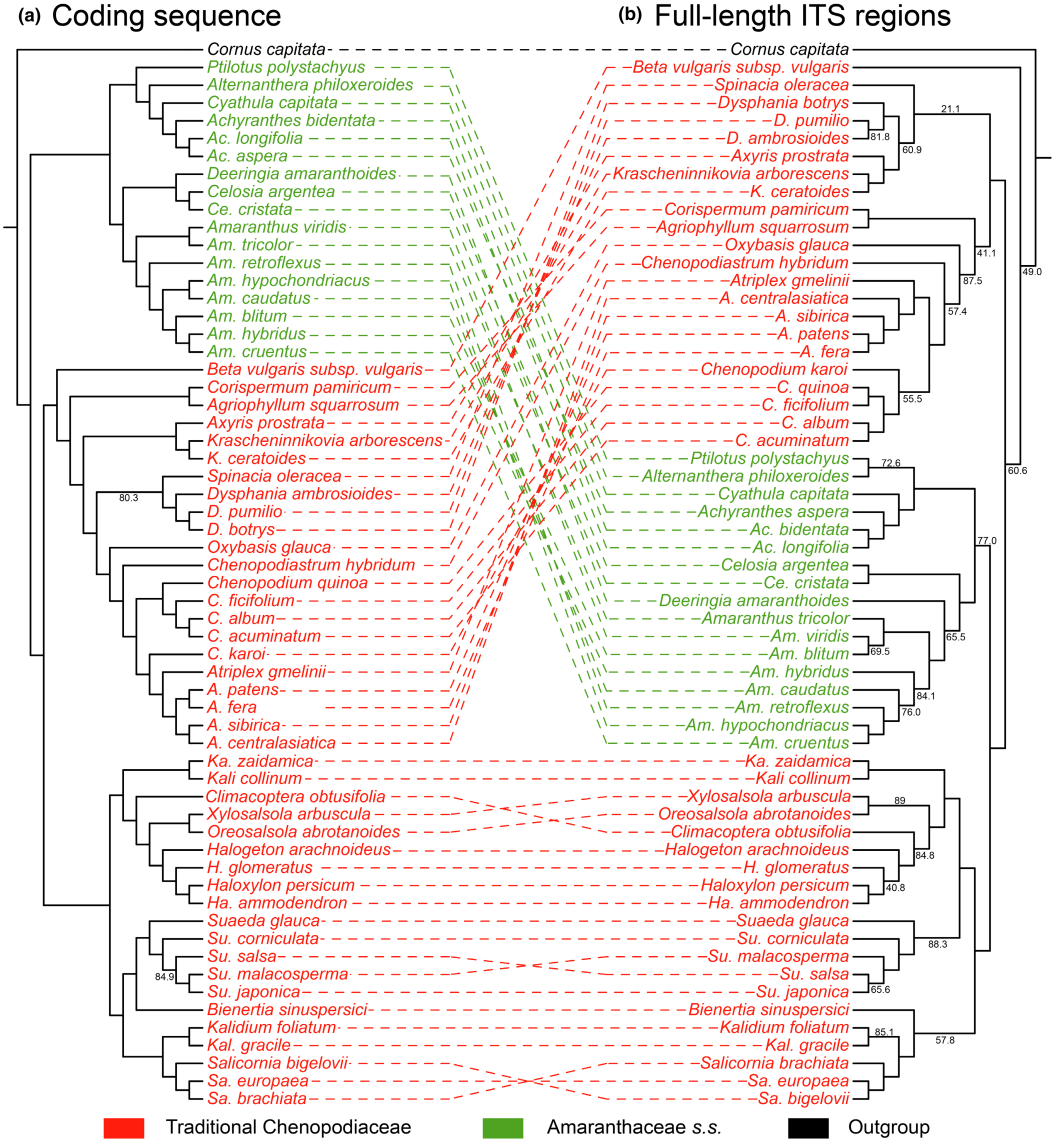
Comparison of the Amaranthaceae *s.l*. plastid and full‐length ITS phylogenies. The topologies of (a) and (b) were reconstructed using maximum likelihood (ML) based on 87 PCGs and full‐length ITS sequences, respectively. The branches are labeled with the values of BS/PP probability, those above 90/0.90 are not displayed. Dotted lines connect the same species between (a) and (b).

To effectively assess the evolutionary information in nucleotides, we used five datasets to determine the phylogenetic relationships within Amaranthaceae *s.l*. using ML and BI statistics. All datasets, except for datasets iii (full‐length ITS) and v (non‐positively selected PCGs), recovered the monophyly of traditional Chenopodiaceae (Clades I and II; Figures 2, S3 and S4). The three instances of clear cytonuclear discordance are as follows: (a) In the PCGs dataset, *C. karoi* is sister to *Atriplex* but is separated from *Atriplex* in the full‐length ITS dataset, (b) *Axyris* L., *Dysphania* R.Br., *Krascheninnikovia*, and *Spinacia* were well resolved in the PCGs dataset as belonging to Chenopodieae, but they were placed independently from Chenopodieae and Corispermeae in the full‐length ITS dataset, and (c) Beteae belonged to Clade I but is the ancestor of Amaranthaceae *s.l*. in the full‐length ITS dataset (Figure 3). The full‐length ITS phylogenetic trees showed relatively low bootstrap values (BS)/posterior probability (PP) in these three discordances. In the Salsoloideae subfamily, *Haloxylon* Bunge is nested in the genera *Halogeton* C. A. Mey., and genera *Halogeton* and *Kali* did not form monophyletic groups.

**FIGURE 4:**
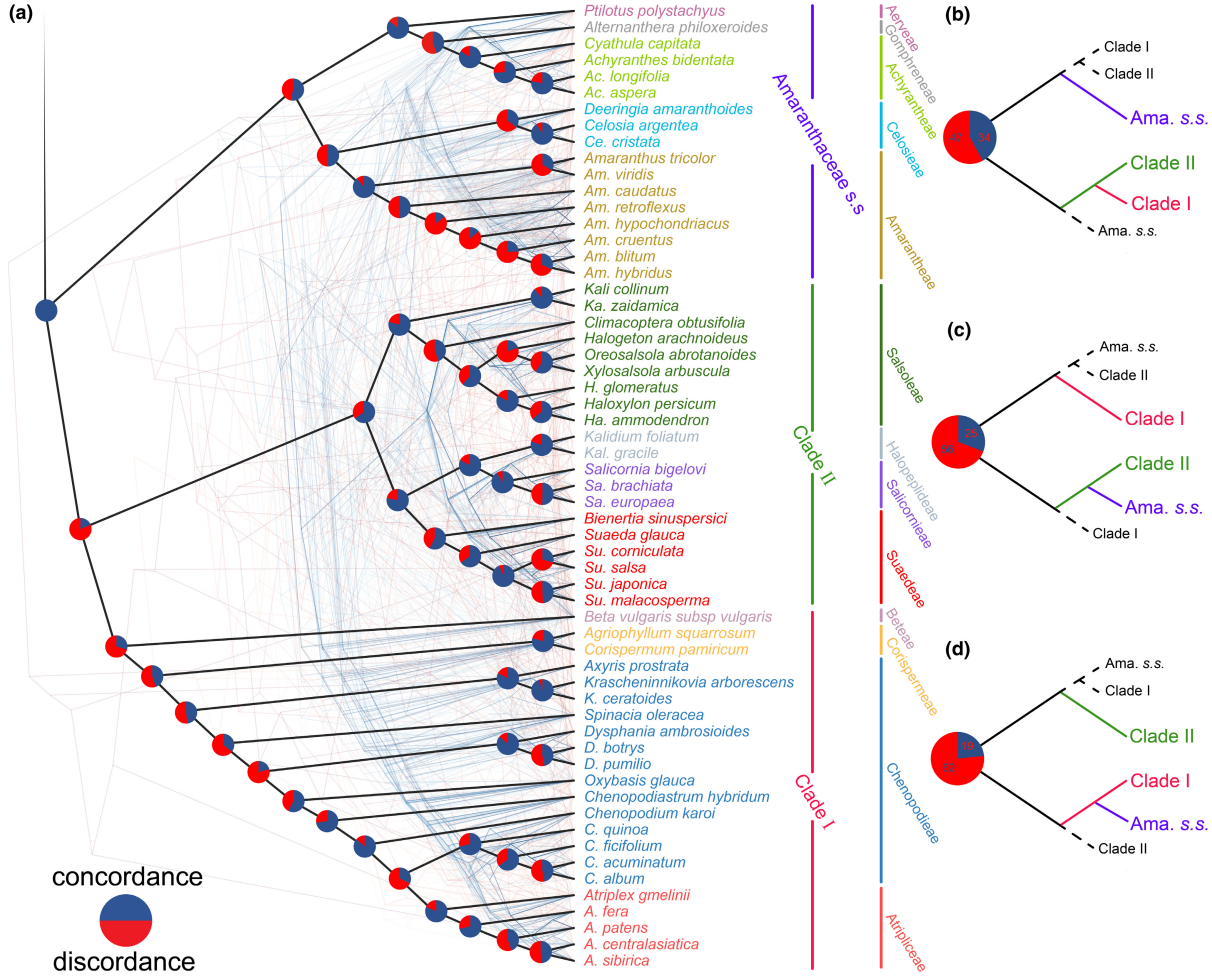
The topology of the species tree based on coalescence shows discordance among 81 shared PCGs (cloud tree). (a) The heavy black line represented the species tree constructed by Astral, and the thin lines represented gene trees constructed by shared PCGs. (b–d) Topology of the three major branches (Clades I, II, and Amaranthaceae *s.s*.) in 81 shared genes of Amaranthaceae *s.l*. Thick‐solid lines indicated branches containing all or most species, while thin‐dotted lines indicated species clustering outside of the branch with few or no members. Three gene trees were not included due to their excessively complicated topology. Pie charts on the nodes showed the degree of concordance between species and gene trees, where the red and blue parts represent discordance and concordance, respectively.

We also compared the coalescent and gene phylogenies, indicating discordance between some gene trees and the species tree within the plastome, as indicated by the Clades of *Atriplex*, *Amaranthus*, Salsoleae, and traditional Chenopodiaceae (Figure 4a). Certain minor Clades (Corispermeae, Salicornieae, Achyrantheae) exhibited high concordance, and their phylogenetic relationships were relatively clear.

### 3.5 Divergence time estimation

**FIGURE 5:**
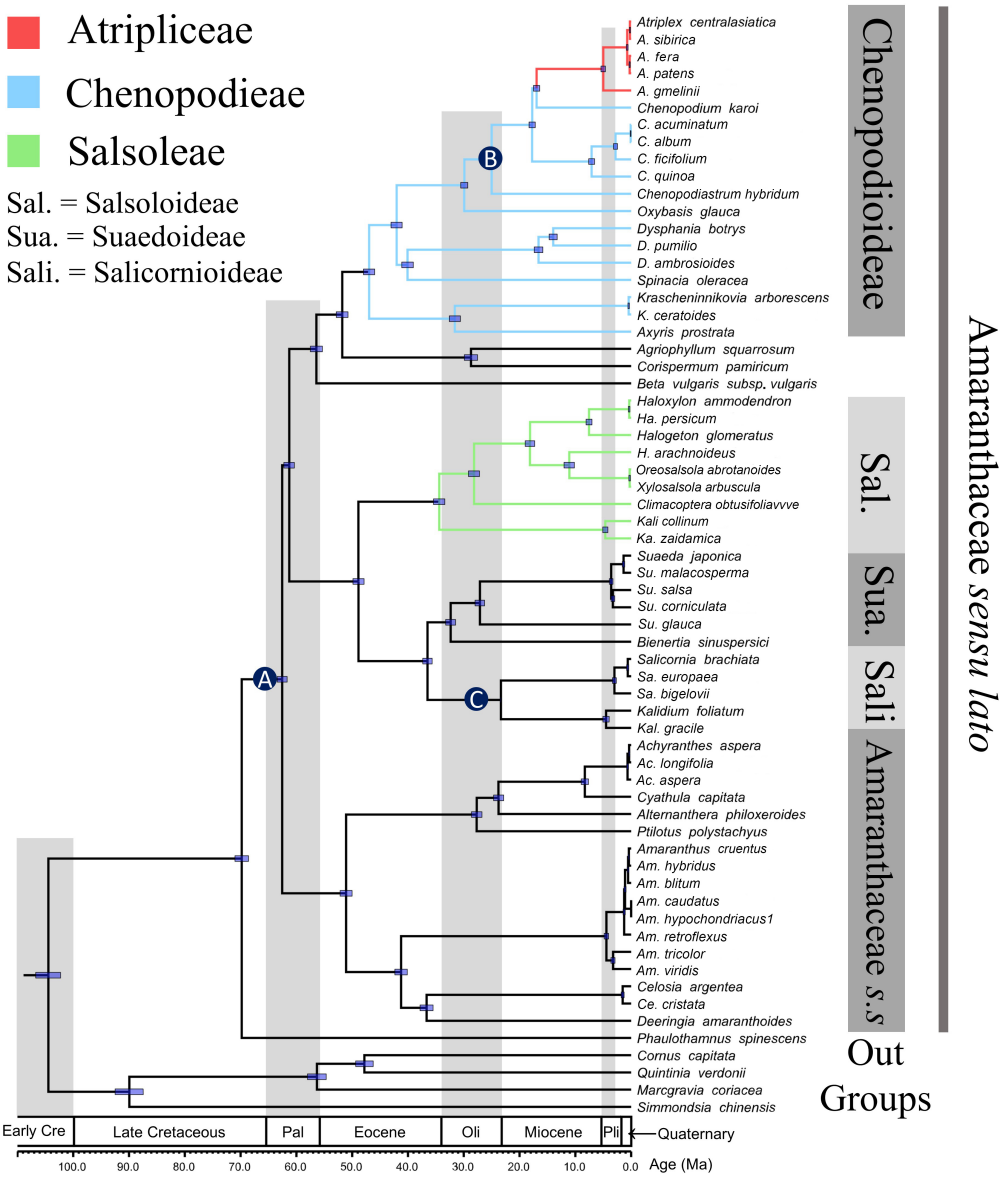
Beast‐derived chronogram of 59 Amaranthaceae *s.l*. using 87 protein‐coding genes. The blue bars correspond to the 95% highest posterior density (HPD) of divergence time, and the blue circles (A = *Polyporina cribraria* Srivastava, B = *Parvangula randeckensis* Hiltermann & Schmitz, C = *Salicornites massalongoi* Principi) represent three fossils to constrain divergence time.

The molecular divergence of Amaranthaceae *s.l*. and its sister taxa Achatocarpaceae (*Phaulothamnus spinescens*) was estimated to have occurred around 69.80 million years ago (Mya) in the late Cretaceous [95% highest posterior density (HPD) = 68.58–71.01 Mya], according to the chronogram based on 87 PCGs and analyzed with BEAST (Figure 5, Table S5). The crown age of Amaranthaceae *s.l*. was estimated to be 62.55 Mya (61.66–63.45 Mya), coinciding with the end of the Cretaceous and the beginning of the Paleocene. During this stage, the former began to differentiate into Clades I, II, and Amaranthaceae *s.s*. around the Cretaceous–Paleogene (K‐Pg) boundary, whereas traditional Chenopodiaceae experienced a rapid differentiation event, mainly reflected in the short differentiation time branches of traditional Chenopodiaceae. The analysis of adaptive evolution indicated that the divergence time of non‐positively selected PCGs was delayed by approximately 20 million years compared to the PCG results. The topology supported the sister relationship of Amaranthaceae *s.s*. to Clade I (Figures S5 and S6). Meanwhile, the results of positively selected genes were consistent with the PCG results and supported the monophyly of traditional Chenopodiaceae.

## 4 DISCUSSION

### 4.1 Gene content and structure of Amaranthaceae *s.l.* plastomes are conserved generally

Accurate identification of plants is vital for the development and breeding of Amaranthaceae *s.l*., not only for protecting genetic resources but also for sustaining the use of diversity (Flowers et al., 2010; Hooper et al., 2005; Myers et al., 2000). In this study, we reported 21 complete plastomes of Amaranthaceae *s.l*., ranging in size from 150,585 to 153,832 bp, which falls within the typical range of angiosperm plastomes (120–180 kb) (Goulding et al., 1996; Zhang et al., 2012). Our published plastome sizes align with previously published plastomes of Amaranthaceae *s.l*. (149,722–155,108 bp; Yao et al., 2019).

Most research has shown that differences in plastome sizes between species are mainly reflected in the boundaries of the IR region (Olmstead & Palmer, 1994). The expansion and contraction of the IR region significantly affect plastome size (Chumley et al., 2006; Zhang et al., 2014). The variation in the IR region length of traditional Chenopodiaceae species tends to be small, indicating that the boundary of the IR region is relatively conserved in the adaptive evolution of Amaranthaceae *s.l*. (Hong et al., 2017). For genes in plastomes, the number and type of rRNA are stable, but there are a few differences in tRNA. Nucleotide mutations in individual species led to the termination of transcription, such as *trn*G^GCC^, *trn*N^GUU^, *trn*S^GCU^, and others (Table 2). However, these differences have little impact on the evolution of species, as they contain minimal evolutionary information. In contrast, variations in the PCG regions can cause changes in the evolutionary information of species. For instance, more than half of Amaranthaceae *s.l*. lack *ycf*15 in the plastomes (Table 2). Comparing the reference sequence of *ycf*15 with plastomes lacking this sequence, we found that gene loss could be due to deletions or substitutions of loci in certain fragments (Figure S7). *Ycf*15 belongs to a family of PCGs that originated from eukaryotes or horizontal transfer (Martin et al., 1998; Stegemann et al., 2012). Our results suggested that this may contribute to differences among plants and may be one reason for the inconsistency between molecular data and morphology.

The arrangement of internal segments in plastomes plays a significant role in multiple evolutionary events during the radiation of Amaranthaceae *s.l*. In *Mammillaria* Haw. plants (Cactaceae) of the Caryophyllales, there was a block of genes rearranged in the LSC region of the plastomes. Although not all *Mammillaria* species had the same rearrangement order, some share the same order with *Carnegiea* Perkins and *Pachycereus* Britton & Rose (both genera belonging to Cactaceae; Solórzano et al., 2019). Through comparative genomic analysis, it was found that there was a 5200 bp reverse complementary fragment in the genera *Chenopodium* L. and *Atriplex* compared to other Amaranthaceae *s.l*. This is similar to the situation we found in the LSC region of *Atriplex* and *Chenopodium*, where the plants of each genus were not completely distinguished in phylogenetic results. This rearrangement block may be a synapomorphy in tribes Atripliceae and Chenopodieae during the radiation evolution of the subfamily Chenopodioideae, suggesting that the arrangement of internal segments of plastomes cannot be ignored.

The plastome is maternally inherited in most angiosperms and is widely used due to its advantages in simple sequencing and assembly, conserved sequences, stable structure, and no interference from genetic recombination (Jansen et al., 2007; Luo et al., 2016). Therefore, hotspots of variation in plastomes are easy to observe, and regions with higher hotspots are often used as molecular markers to help correctly distinguish taxon and effectively protect wild plant resources. To analyze the nucleotide diversity of 79 de‐redundant PCGs and 127 intergenic spacer regions of 59 species of Amaranthaceae *s.l*., we employed DnaSP and mVISTA to compare the complete plastomes. The results showed that changes in the intergenic spacer regions are significantly greater than those in the coding regions (Figures 1 and S2). This is consistent with previous studies using short orthologous DNA sequences (DNA barcodes) containing evolutionary information to analyze the phylogeny of Amaranthaceae *s.l*. (Kress et al., 2005). However, these studies have some problems, such as low support rates and difficulties in effectively distinguishing species. The reason for these issues may be that the nucleotide diversity of loci such as *rbc*L (0.0414), *atp*B‐*rbc*L (0.0738), and *psb*B‐*psb*H (0.0766) used in prior research is lower than the average nucleotide diversity of PCGs (0.05) and intergenic spacer regions (0.0829), respectively (Kadereit et al., 2003, 2010; Kadereit & Freitag, 2011; Schüssler et al., 2017). Therefore, the diversity hotspots obtained in this study can provide insights on how to better classify species with controversial classification within Amaranthaceae *s.l*.

### 4.2 Adaptive evolution

During the evolution of Amaranthaceae *s.l*., we identified eight positively selected genes. *Ycf*1 and *ycf*2 encode components of the plastome inner envelope membrane protein, crucial for cell survival. They are also the two largest open reading frames in plastomes (Drescher et al., 2000; Kikuchi et al., 2013). In the plastomes of Amaranthaceae *s.l*. examined in this study, *ycf*1 gene spans approximately 5400 bp, extending across the SSC and IR regions. Due to a small segment of *ycf*1 (approximately 1000 bp) being located on the IR side, its reverse complementary property results in a 1000 bp *ycf*1 pseudogene within another IR region. This pseudogene is only 1/5 the length of *ycf*1 due to premature stop codons, ultimately resulting in its lack of functional characteristics of *ycf*1. Prior research has reported strong positive selection effects of these two genes in *Quercus* and *Caragana* (Jiang et al., 2018; Yang et al., 2016). Among other positive selected loci, *acc*D, a gene encoding the β‐carboxyl transferase subunit of acetyl‐CoA carboxylase, maintains the plastome compartment and has ACCase that synthesizes substances necessary for leaf development (Kode et al., 2005). Additionally, *ccs*A encodes a protein required for heme attachment to c‐cytochromes (Xie & Merchant, 1996). Thus, these four genes improve the oxidation of Amaranthaceae *s.l*. plant cells in extreme environments by better‐utilizing oxygen. The remaining positively selected genes have more or less beneficial effects on plant cells. Environment selection enables species to adapt to complex and variable habitats. Previous studies have also reported positive selection of these genes in plants such as *Gentiana* L., Bignoniaceae, and Amaranthaceae, which have adapted to adversity, evolving characteristics suitable for surviving in harsh environments (Kapralov et al., 2012; Sobreiro et al., 2020; Zhou et al., 2018).

Intriguingly, the branch‐site models, with traditional Chenopodiaceae as the foreground branch, did not detect any positively selected amino acid sites in *ccs*A, and its LRT‐test results were not particularly significant (.01 < *p* < .05, Table S4). The positively selected sites in the remaining seven genes were highly similar to the site model results, with significant differences in LRT‐test and *ω* > 1. The phylogenetic topology of these seven positively selected genes mainly formed ((Clades I and II), Amaranthaceae *s.s*., Table S6), suggesting that they may be one of the driving forces for the evolution of traditional Chenopodiaceae into a monophyletic group. The adaptive evolution of these seven genes in traditional Chenopodiaceae Clade has played an important role, providing insight into the adaptive evolution of Amaranthaceae *s.l*. and further elucidating chloroplast genetic characteristics.

*Ycf*15 is a gene in plastomes that has drawn much attention from researchers due to its paradoxical function and evolution (Chumley et al., 2006; Raubeson et al., 2007). Shi et al. explored the function and evolution of the *ycf*15 gene in angiosperms based on plastomes of *Camellia* L. species and suggested that *ycf*15 contains certain phylogenetic information sites causing the divergence topology of branch ends (Shi et al., 2013). However, there is no direct molecular evidence currently suggesting that *ycf*15 is involved in adaptive evolution. In this study, we mapped the presence of *ycf*15 onto the phylogenetic tree of Amaranthaceae *s.l*. (Figure 2). Our results indicate that all *Atriplex* plants lack *ycf*15, and all *Chenopodium* plants contain *ycf*15 except for *C. karoi*, which is the closest genus to *Atriplex*. Similarly, in the Salsoloideae Clade, the plastomes of *Halogeton arachnoideus*, *Haloxylon persicum*, *Kali zaidamicum*, and *Climacoptera obtusifolia* include *ycf*15, but its closely related species do not. The species were collected from areas with severe climatic conditions. The saline‐alkali environment, water shortage, and strong sunlight may limit the growth of halophytes, and the genes may evolve in response to the environment, forming characteristics that differ from the related species (Cheng et al., 2021; Guan et al., 2015; Tao et al., 2017). In addition, the absence of *ycf*15 within the plastomes of Amaranthaceae *s.s*. is one of the features that distinguish these plants from traditional Chenopodiaceae. This study speculates that *ycf*15 in Amaranthaceae *s.l*. may undergo variation in response to extreme environments.

### 4.3 Phylogeny and molecular dating analysis of Amaranthaceae *s.l.*

Previous researchers have conducted extensive molecular systematics studies on Amaranthaceae *s.l*. (Hernández‐Ledesma et al., 2015; Kadereit et al., 2003). However, controversies still exist regarding the systematic relationships between the main lineages of the family (Hammer et al., 2015; Masson & Kadereit, 2013). Based on concatenated PCGs, our study shows that traditional Chenopodiaceae is a monophyly (Clades I and II), while Amaranthaceae *s.s*. forms an independent clade and is a sister taxon to traditional Chenopodiaceae (Figure 2). Coalescence‐based simulation plots revealed discrepancies between some phylogenetic topologies of cloud trees and species tree trees (Figure 4a). Different genes within species may evolve diversely, leading to a large number of ILS events, such as ITS trees with low support rates (Figure 3). Although a few gene trees support Clades I and II as sister groups, their support rates are high. Statistical analysis of the topology of these gene trees (Figure 4b–d, Table S6) shows that several Amaranthaceae *s.s*. species clustered within traditional Chenopodiaceae or a small number of traditional Chenopodiaceae species clustered within Amaranthaceae *s.s*., but this did not affect the primary topology ((Clades I and II), Amaranthaceae *s.s*.), which is supported by most gene trees. This provides compelling molecular evidence that traditional Chenopodiaceae constitutes a monophyletic group, which contradicts the notion that traditional Chenopodiaceae and Amaranthaceae *s.s*. should be included in Amaranthaceae *s.l*. based on APG IV.

Yao et al. (2019) proposed, based on the combined‐complete matrix of Caryophyllales plastid PCGs, that traditional Chenopodiaceae was not a monophyletic lineage. However, the samples did not cover representative tribes in Amaranthaceae *s.l*. (Atripliceae, Chenopodieae, Salsoleae, etc.) and had low support (BS = 69%; Yao et al., 2019). The phylogeny of Amaranthaceae *s.l*. based on eight plastid DNA fragments also echoed Yao's conclusion (Huang et al., 2020).

Transcriptomes help infer species trees and reveal evolutionary complexity in lineages beyond what is possible with DNA fragments alone. Yang et al. (2018) demonstrated that traditional Chenopodiaceae was a monophyletic group closely related to Amaranthaceae *s.s*. using transcriptome and genome data based on homology inference methods. Walker et al. (2018) analyzed gene tree conflicts using transcriptome data and gained similar results to Yang et al. Their findings showed that conserved molecular sequences containing phylogenetic information, such as coding sequences, are valuable for revealing evolutionary relationships. Additionally, our results are consistent with the above‐mentioned studies in terms of intergroup relationships, and plastid PCGs are more conservative than transcriptomes, which can enhance the results of Yang and Walker's studies.

For Clade I, the primary taxonomic contradiction lies between the genera *Chenopodium* and *Atriplex* based on concatenated phylogeny. Our results indicate that, compared to *Chenopodium*, the relationships between *C. karoi* and *Atriplex* are closer, and *C. hybridum* (L.) is the ancestral species of the two genera (*Atriplex* and *Chenopodium*). However, the phylogenetic position of these two species remains ambiguous in the analyses based on both coalescent simulation and full‐length ITS (Figure 3). Thus, *Chenopodium* is not a monophyletic group due to the existence of *C. karoi*, which is strongly supported in most phylogenetic trees (Figures 4, S3 and S4). The classification of the genera *Chenopodium* and *Atriplex* has been inconsistent in taxonomical history. It was suggested that *Chenopodium s.l*. should be divided into at least three independent lineages in the Chenopodiaceae–Amaranthaceae alliance (Kadereit et al., 2003; Müller & Borsch, 2005). In a study based on *trn*L‐F and ITS, five independent lineages of Chenopodioideae had high support values, but the classification positions of *Chenopodiastrum hybridum* (L.) S. Fuentes, Uotila & Borsch and *Oxybasis* Kar. & Kir. were still controversial, leading researchers to suggest the establishment of additional lineages or merging Atripliceae and Chenopodieae (Fuentes‐Bazan, Mansion, & Borsch, 2012). Another study based on *trn*L‐F and *mat*K suggested that the four branches of Chenopodioideae and the core group of *Chenopodium* should form a monophyletic group called the tribe Atripliceae *s.l*. (this genus name has priority over Chenopodieae; Fuentes‐Bazan, Uotila, & Borsch, 2012). Morphologically, *C. karoi* differs from the genus *Chenopodium* in having leaves abaxially with white powder and seed surface with honeycomb pits. We cannot evaluate the taxonomic status of *C. karoi* due to the lack of sufficient plastome samples, but we suggest separating *C. karoi* from the genus *Chenopodium*.

For subfamily Salsoloideae in Clade II, we found that the genera *Halogeton* and Subtr. Salsolinae are not monophyletic lineage. Akhani et al. divided the conflicted taxonomic relationships within *Salsola* into multiple genera based on molecular and morphological information (Akhani et al., 2007). Wen et al. proposed that *Halogeton* was polyphyletic, and *Kali collinum* and *Ka. zaidamica* were not in the same clade as *Xylosalsola arbuscula* (Pall.) based on ITS and plastome fragments (Wen et al., 2010). Our phylogenetic results also verified that Subtr. Salsolinae was not a monophyletic lineage (Figure 4). The Subtr. Salsolinae species may have developed certain morphological characteristics to adapt repeatedly to drought and saline environments through radiation evolution, such as central sclerenchyma and reduction of surface area in leaves, which promote their C4 cycle and improve productivity under arid conditions (Lauterbach et al., 2017; Schüssler et al., 2017). Therefore, it is recommended that Subtr. Salsolinae should be split into multiple subfamilies to conform to correct taxonomic classification.

### 4.4 Divergence of estimation of Amaranthaceae *s.l.*

We provide the origin and molecular evolution time of Amaranthaceae *s.l*. based on plastomes, positively selected genes, and non‐positively selected genes, showing that the stem divergence time of Amaranthaceae *s.l*. occurred during the Paleogene period around 69.80 Mya near the K‐Pg boundary (Figures 5, S5 and S6, Table S5). This estimate is approximately the median value of previous studies, which estimated the origin time of Amaranthaceae *s.l*. using *rbc*L and *atp*B‐*rbc*L internal spacer regions (47–87 Mya; Kadereit et al., 2012; Magallón et al., 2015). Magallón et al. used five plastid and nuclear markers to conclude that the stem age of Amaranthaceae *s.l*. occurred at 64.2 or 76.4 Mya (Kadereit et al., 2012; Magallón et al., 2015). Moreover, it was interesting to note that positively selected genes played a driving role in the evolution of Amaranthaceae *s.l*. The divergence time tree based on these genes confirms the monophyly of traditional Chenopodiaceae and explains the presence of numerous ILS events within the plastome that have previously hindered accurate inference of Amaranthaceae *s.l*. phylogeny.

We also found that Clades I and II differentiated rapidly in the early Paleocene (61.27 Mya), indicating that broad‐scale evolutionary patterns were evident within a clade radiation. The extinction event at the end of the Cretaceous led to the death of many organisms due to their inability to adapt to drastic climate change, thus allowing the surviving plants to reproduce rapidly and occupy available niches (Fawcett et al., 2009; Meredith et al., 2011; Schulte et al., 2010). During periods of less than one Mya or two to three Mya, the Qaidam Basin experienced several stages of the penultimate glacial maximum, last glacial maximum, and interglacial periods (Owen et al., 2006; Schäfer et al., 2002). As the warm period of the interglacial period arrived, Amaranthaceae *s.l*. started to disperse from refugia to find their niche for adaptive evolution. Species of the same genus also experienced changes in molecular and morphological characteristics to cope with local climate characteristics, leading to the subsequent evolution of Amaranthaceae *s.l*. plants in various directions.

## 5 CONCLUSIONS

We sequenced and reported the plastomes of 20 species of Amaranthaceae *s.l*. collected from the Qaidam Basin and one from Gaize County in Tibet Autonomous Region. Using coalescence‐based and concatenated plastid PCGs, we provided strong evidence for the monophyly of traditional Chenopodiaceae and its sister group relationship with Amaranthaceae *s.s*. Phylogeny reconstruction of plastomes and full‐length ITS revealed discordance and cytonuclear conflict in the gene trees, likely due to ILS events resulting from differences in climate environments among species in Amaranthaceae *s.l*. Additionally, phylogenetic analysis of adaptive evolution demonstrated that positively selected genes played a driving role in the evolutionary direction of Amaranthaceae *s.l*. Regarding the taxonomic relationships within tribes, our results showed that Chenopodieae is a non‐monophyletic group. To address this, we suggest taking the following steps: (1) establishing additional tribes or merging Chenopodieae and Atripliceae into one tribe and (2) separating *C. karoi* from the genus Chenopodieae. *Halogeton* and Subtr. Salsolinae are also paraphyletic groups, which is consistent with prior research. The formation of lineages within Amaranthaceae *s.l*. can be diverse, with environmental stress pushing the genes to move in adaptive directions, such as in the case of the deletion of *ycf*15. In addition, our molecular time estimation confirmed that the biological extinction events in the Cretaceous period led to the evolution of Amaranthaceae *s.l*. into three branches: Clades I, II, and Amaranthaceae *s.s*. Once the plants found suitable niches, they began to further differentiate during the cycle of the last glacial, which is strongly consistent with the expected results of previous biogeographic studies.

## AUTHOR CONTRIBUTIONS

**Hao Xu:** Data curation (lead); formal analysis (lead); visualization (lead); writing – original draft (lead); writing – review and editing (equal). **Yuqin Guo:** Project administration (equal). **Mingze Xia:** Visualization (equal). **Jingya Yu:** Supervision (equal). **Xiaofeng Chi:** Software (equal). **Yun Han:** Conceptualization (equal). **Xiaoping Li:** Resources (equal). **Faqi Zhang:** Funding acquisition (equal); writing – review and editing (equal).

## CONFLICT OF INTEREST STATEMENT

The authors have no conflicts of interest to declare.

## Supporting information


Data S1.

## Data Availability

All assembly data have been deposited at the NCBI. Plastomes: GenBank accession numbers NCBI: MZ230595, ON149841–ON149860. Full‐length ITS: OQ581542–OQ581562.
