# Supplementary material for: An updated phylogeny and adaptive evolution within Amaranthaceae s.l. inferred from multiple phylogenomic datasets
Source: Ecol Evol. 2024 Jul 14;14(7):e70013. doi: 10.1002/ece3.70013 (PMC11246835; doi:10.1002/ece3.70013)
Supplement: Supplementary file 1 — Data S1. [file ECE3-14-e70013-s001.docx]

**Table S1** The basic plastid genome characteristics of 59 Amaranthaceae *s.l.* and 5 out groups.

|  | **Size (bp)** | | | |  | **Number** | | |  |  |
| --- | --- | --- | --- | --- | --- | --- | --- | --- | --- | --- |
| **Taxon** | **Genome** | **LSC** | **SSC** | **IR** |  | **PCGs** | **tRNA** | **rRNA** | **GC (%)** | **Accession No.** |
| **Chenopodioideae** |  |  |  |  |  |  |  |  |  |  |
| Atripliceae |  |  |  |  |  |  |  |  |  |  |
| *Atriplex* *centralasiatica* | 152 237 | 83 721 | 18 096 | 25 210 |  | 85 | 37 | 8 | 37.3 | NC_045304.1 |
| *Atriplex* *gmelinii* | 151 852 | 83 872 | 17 812 | 25 084 |  | 85 | 37 | 8 | 37.3 | NC_059062 |
| *Atriplex* *patens* | 152 221 | 83 714 | 18 097 | 25 205 |  | 85 | 37 | 8 | 37.3 | ON149850 |
| *Atriplex sibirica* | 152 220 | 83 711 | 18 089 | 25 210 |  | 85 | 37 | 8 | 37.3 | ON149852 |
| *Atriplex fera* | 152 203 | 83 703 | 18 090 | 25 205 |  | 85 | 37 | 8 | 37.3 | ON149857 |
| Chenopodieae |  |  |  |  |  |  |  |  |  |  |
| *Axyris prostrata* | 151 353 | 83 122 | 18 033 | 25 099 |  | 85 | 37 | 8 | 37.1 | ON149854 |
| *Chenopodiastrum hybridum* | 152 948 | 84 234 | 18 396 | 25 159 |  | 87 | 37 | 8 | 37.1 | ON149849 |
| *Chenopodium* *acuminatum* | 152 200 | 83 683 | 18 131 | 25 193 |  | 87 | 37 | 8 | 37.2 | NC_054154 |
| *Chenopodium* *album* | 152 167 | 83 676 | 18 105 | 25 193 |  | 87 | 37 | 8 | 37.2 | MW417304.1 |
| *Chenopodium* *ficifolium* | 151 823 | 83 668 | 17 937 | 25 109 |  | 87 | 37 | 8 | 37.3 | NC_041200.1 |
| *Chenopodium karoi* | 152 636 | 82 620 | 18 150 | 25 933 |  | 85 | 37 | 8 | 37.6 | ON149853 |
| *Chenopodium* *quinoa* | 152 079 | 83 553 | 18 118 | 25 204 |  | 87 | 37 | 8 | 37.2 | MK159176 |
| *Dysphania ambrosioides* | 151 689 | 83 421 | 18 062 | 25 103 |  | 87 | 37 | 8 | 36.9 | NC_041201.1 |
| *Dysphania botrys* | 152 055 | 83 769 | 17 916 | 25 185 |  | 87 | 37 | 8 | 36.9 | NC_042166.1 |
| *Dysphania pumilio* | 151 960 | 83 756 | 17 742 | 25 231 |  | 87 | 37 | 8 | 36.9 | MK541016.1 |
| *Krascheninnikovia arborescens* | 151 832 | 86 688 | 18 984 | 23 080 |  | 85 | 37 | 8 | 36.6 | ON149851 |
| *Krascheninnikovia ceratoides* | 152 181 | 84 082 | 19 295 | 24 402 |  | 85 | 37 | 8 | 36.6 | ON149841 |
| *Oxybasis glauca* | 151 655 | 83 759 | 17 914 | 24 991 |  | 87 | 37 | 8 | 36.9 | NC_047226.1 |
| *Spinacia oleracea* | 150 725 | 82 719 | 17 860 | 25 073 |  | 87 | 37 | 8 | 36.8 | NC_002202.1 |
| Corispermeae |  |  |  |  |  |  |  |  |  |  |
| *Agriophyllum squarrosum* | 151 759 | 85 001 | 20 014 | 23 372 |  | 85 | 37 | 8 | 36.7 | ON149847 |
| *Corispermum pamiricum* | 150 614 | 84 221 | 19 741 | 23 326 |  | 85 | 37 | 8 | 36.5 | ON149842 |
| **Salicornioideae** |  |  |  |  |  |  |  |  |  |  |
| Halopeplideae |  |  |  |  |  |  |  |  |  |  |
| *Kalidium foliatum* | 153 832 | 84 845 | 18 989 | 24 999 |  | 85 | 36 | 8 | 36.3 | ON149848 |
| *Kalidium gracile* | 153 646 | 84 770 | 18 928 | 24 974 |  | 85 | 37 | 8 | 36.3 | ON149858 |
| Salicornieae |  |  |  |  |  |  |  |  |  |  |
| *Salicornia bigelovii* | 153 076 | 84 485 | 18 947 | 24 822 |  | 87 | 37 | 8 | 36.3 | NC_027226.1 |
| *Salicornia brachiata* | 153 324 | 84 616 | 18 820 | 24 944 |  | 87 | 37 | 8 | 36.2 | NC_027224.1 |
| *Salicornia europaea* | 153 232 | 84 573 | 18 855 | 24 902 |  | 87 | 37 | 8 | 36.2 | NC_027225.1 |
| **Salsoloideae** |  |  |  |  |  |  |  |  |  |  |
| Salsoleae |  |  |  |  |  |  |  |  |  |  |
| *Climacoptera obtusifolia* | 152 933 | 84 015 | 18 580 | 25 169 |  | 87 | 37 | 8 | 36.6 | ON149859 |
| *Halogeton arachnoideus* | 152 773 | 84 796 | 18 859 | 24 559 |  | 87 | 37 | 8 | 36.7 | ON149845 |
| *Halogeton glomeratus* | 151 404 | 83 529 | 18 965 | 24 455 |  | 85 | 37 | 8 | 36.4 | ON149846 |
| *Haloxylon ammodendron* | 151 570 | 84 214 | 19 014 | 24 171 |  | 86 | 37 | 8 | 36.5 | ON149844 |
| *Haloxylon persicum* | 151 586 | 84 217 | 18 943 | 24 213 |  | 87 | 37 | 8 | 36.6 | NC_027669.1 |
| *Kali collinum* | 150 585 | 83 974 | 18 349 | 24 131 |  | 85 | 37 | 8 | 36.4 | ON149855 |
| *Kali zaidamicum* | 150 738 | 84 092 | 18 404 | 24 121 |  | 87 | 37 | 8 | 36.4 | MZ230595 |
| *Oreosalsola abrotanoides* | 151 613 | 84 646 | 19 565 | 23 701 |  | 85 | 37 | 8 | 36.8 | ON149856 |
| *Xylosalsola arbuscula* | 151 614 | 84 651 | 19 561 | 23 701 |  | 85 | 37 | 8 | 36.8 | ON149843 |
| **Suaedoideae** |  |  |  |  |  |  |  |  |  |  |
| Suaedeae |  |  |  |  |  |  |  |  |  |  |
| *Bienertia sinuspersici* | 153 472 | 84 604 | 19 016 | 24 926 |  | 86 | 37 | 8 | 36.6 | KU726550.2 |
| *Suaeda corniculata* | 151 714 | 83 209 | 18 101 | 25 202 |  | 85 | 37 | 8 | 36.4 | ON149860 |
| *Suaeda glauca* | 149 807 | 82 162 | 18 191 | 24 727 |  | 87 | 37 | 8 | 36.5 | NC_045303.1 |
| *Suaeda japonica* | 152 109 | 83 618 | 18 101 | 25 195 |  | 87 | 37 | 8 | 36.3 | NC_042675.1 |
| *Suaeda malacosperma* | 151 989 | 83 492 | 18 121 | 25 188 |  | 87 | 37 | 8 | 36.3 | NC_039180.1 |
| *Suaeda salsa* | 151 642 | 83 502 | 17 780 | 25 180 |  | 87 | 37 | 8 | 36.4 | NC_045302.1 |
| **Amaranthoideae** |  |  |  |  |  |  |  |  |  |  |
| Achyrantheae |  |  |  |  |  |  |  |  |  |  |
| *Achyranthes aspera* | 151 445 | 83 893 | 17 252 | 25 150 |  | 85 | 37 | 8 | 36.4 | MW411185.1 |
| *Achyranthes bidentata* | 151 441 | 83 890 | 17 261 | 25 145 |  | 85 | 37 | 8 | 36.5 | MN255842.1 |
| *Achyranthes longifolia* | 151 520 | 83 966 | 17 254 | 25 150 |  | 85 | 36 | 8 | 36.4 | NC_050061.1 |
| *Cyathula capitata* | 151 557 | 83 352 | 17 127 | 25 539 |  | 84 | 37 | 8 | 36.4 | NC_041262.1 |
| Aerveae |  |  |  |  |  |  |  |  |  |  |
| *Ptilotus polystachyus* | 150 002 | 82 783 | 17 313 | 24 953 |  | 85 | 37 | 8 | 36.5 | NC_046575.1 |
| Amarantheae |  |  |  |  |  |  |  |  |  |  |
| *Amaranthus blitum* | 150 775 | 84 083 | 17 990 | 24 351 |  | 85 | 37 | 8 | 36.6 | MW255966.1 |
| *Amaranthus caudatus* | 150 523 | 83 878 | 17 941 | 24 352 |  | 85 | 37 | 8 | 36.6 | NC_040143.1 |
| *Amaranthus hybridus* | 150 759 | 84 103 | 17 954 | 24 351 |  | 85 | 37 | 8 | 36.6 | NC_053787.1 |
| *Amaranthus cruentus* | 150 757 | 84 101 | 17 954 | 24 351 |  | 85 | 37 | 8 | 36.6 | MG836507.1 |
| *Amaranthus hypochondriacus* | 150 523 | 83 878 | 17 941 | 24 352 |  | 85 | 37 | 8 | 36.6 | MG836505.1 |
| *Amaranthus retroflexus* | 150 710 | 83 892 | 18 100 | 24 359 |  | 85 | 37 | 8 | 36.6 | MW646089.1 |
| *Amaranthus tricolor* | 150 027 | 83 735 | 17 600 | 24 346 |  | 84 | 37 | 8 | 36.6 | KX094399.1 |
| *Amaranthus viridis* | 150 452 | 83 832 | 17 914 | 24 353 |  | 85 | 37 | 8 | 36.6 | MW679034.1 |
| Celosieae |  |  |  |  |  |  |  |  |  |  |
| *Celosia argentea* | 153 673 | 85 140 | 17 711 | 25 411 |  | 85 | 37 | 8 | 36.8 | NC_041294.1 |
| *Celosia cristata* | 153 472 | 84 975 | 17 681 | 25 408 |  | 85 | 37 | 8 | 36.7 | NC_045887.1 |
| *Deeringia amaranthoides* | 155 108 | 86 067 | 18 303 | 25 369 |  | 85 | 35 | 8 | 36.8 | NC_041267 |
| Gomphreneae |  |  |  |  |  |  |  |  |  |  |
| *Alternanthera philoxeroides* | 152 255 | 84 670 | 17 319 | 25 133 |  | 85 | 36 | 8 | 36.4 | MW285080.1 |
| **Betoideae** |  |  |  |  |  |  |  |  |  |  |
| Beteae |  |  |  |  |  |  |  |  |  |  |
| *Beta vulgaris* subsp. *vulgaris* | 149 722 | 117 123 | 17 793 | 7 403 |  | 85 | 35 | 8 | 37.0 | KR230391.1 |
| **Out groups** |  |  |  |  |  |  |  |  |  |  |
| *Cornus capitata* | 157 200 | 86 564 | 18 412 | 26 112 |  | 85 | 37 | 8 | 38.2 | MG524998.1 |
| *Marcgravia coriacea* | 158 401 | 87 262 | 18 629 | 26 255 |  | 85 | 37 | 8 | 36.7 | NC_041255.1 |
| *Phaulothamnus spinescens* | 156 987 | 86 964 | 18 885 | 25 569 |  | 87 | 37 | 8 | 36.9 | MH286322 |
| *Quintinia verdonii* | 157 371 | 86 805 | 18 730 | 25 918 |  | 84 | 37 | 8 | 38.2 | NC_041281.1 |
| *Simmondsia chinensis* | 151 737 | 86 206 | 17 307 | 24 112 |  | 85 | 37 | 8 | 36.8 | NC_040935.1 |

**Table S2** Accession of full-length ITS of Amaranthaceae *s.l.*

| **Taxon** | **Accession** |
| --- | --- |
| *Achyranthes aspera* | MT112918.1 |
| *Achyranthes bidentata* | DQ813300.1 |
| *Achyranthes longifolia* | MG730615.1 |
| *Agriophyllum squarrosum* | OQ581549 |
| *Alternanthera philoxeroides* | KY968872.1 |
| *Amaranthus blitum* | KY968874.1 |
| *Amaranthus caudatus* | MG685215.1 |
| *Amaranthus hybridus* | KY968887.1 |
| *Amaranthus cruentus* | KR150154.1 |
| *Amaranthus hypochondriacus* | KY968929.1 |
| *Amaranthus retroflexus* | MH711405.1 |
| *Amaranthus tricolor* | KY968890.1 |
| *Amaranthus viridis* | KY968863.1 |
| *Atriplex centralasiatica* | DQ086481.1 |
| *Atriplex fera* | OQ581559 |
| *Atriplex gmelinii* | HM587507.1 |
| *Atriplex patens* | OQ581552 |
| *Atriplex sibirica* | OQ581554 |
| *Axyris prostrata* | OQ581556 |
| *Beta vulgaris subsp. vulgaris* | LK054241.1 |
| *Bienertia sinuspersici* | DQ499349.1 |
| *Celosia argentea* | KY968928.1 |
| *Celosia cristata* | KY968942.1 |
| *Chenopodium acuminatum* | MH768090.1 |
| *Chenopodium album* | MH711165.1 |
| *Chenopodium ficifolium* | KY968936.1 |
| *Chenopodium hybridum* | OQ581551 |
| *Chenopodiastrum hybridum* | OQ581555 |
| *Chenopodium quinoa* | KF709219.1 |
| *Corispermum pamiricum* | OQ581543 |
| *Cornus capitata* | MH710997.1 |
| *Cyathula capitata* | LT995188.1 |
| *Deeringia polysperma* | LC604844.1 |
| *Dysphania ambrosioides* | KY968860.1 |
| *Dysphania botrys* | MK802964.1 |
| *Dysphania pumilio* | KY968827.1 |
| *Halogeton arachnoideus* | OQ581547 |
| *Halogeton glomeratus* | OQ581548 |
| *Haloxylon ammodendron* | OQ581546 |
| *Haloxylon persicum* | EF453438.1 |
| *Kalidium foliatum* | OQ581550 |
| *Kalidium gracile* | OQ581560 |
| *Krascheninnikovia arborescens* | OQ581553 |
| *Krascheninnikovia ceratoides* | OQ581542 |
| *Oxybasis glauca* | KY968937.1 |
| *Ptilotus polystachyus* | KP875951.1 |
| *Salicornia bigelovii* | EU682686.1 |
| *Salicornia brachiata* | JQ341058.1 |
| *Salicornia europaea* | AY489247.1 |
| *Oreosalsola abrotanoides* | OQ581558 |
| *Xylosalsola arbuscula* | OQ581544 |
| *Kali collinum* | OQ581557 |
| *Kali zaidamicum* | OQ581545 |
| *Climacoptera obtusifolia* | OQ581561 |
| *Spinacia oleracea* | AB935678.1 |
| *Suaeda corniculata* | OQ581562 |
| *Suaeda glauca* | MW004660.1 |
| *Suaeda japonica* | MT875064.1 |
| *Suaeda malacosperma* | DQ786337.1 |
| *Suaeda salsa* | MW004654.1 |

**Table S3** Results of maximum likelihood parameters and test hypotheses for 79 de-redundant PCGs with evidence of positively selected sites based on the site models.

| **Gene** | **Model** | **np** | **lnL** | **Estimates of parameters** | | | **Positively selected sites** |
| --- | --- | --- | --- | --- | --- | --- | --- |
| *acc*D | M1 | 118 | -7,342.19 | | p= 0.75327 0.24673 | w= 0.09219 1.00000 |  |
|  | M2 | 120 | -7,308.83 | | p= 0.73178 0.23720 0.03102 | w= 0.09643 1.00000 4.26656 | 58 Q 0.999** 130 E 0.997** 145 R 0.999** 291 P 1.000** 407 G 0.999** |
|  | M7 | 118 | -7,343.94 | | p = 0.24741 q = 0.61908 | |  |
|  | M8 | 120 | -7,302.85 | | p0 = 0.96089 p = 0.33472 q = 0.98622 | (p1 = 0.03911) w = 3.41634 | 58 Q 1.000** 130 E 0.999** 145 R 1.000** 291 P 1.000** 407 G 0.999** |
| *atp*A | M1 | 118 | -6,652.95 | | p= 0.95523 0.04477 | w= 0.02616 1.00000 |  |
|  | M2 | 120 | -6,652.95 | | p= 0.95523 0.04477 0.00000 | w= 0.02616 1.0000 21.76585 |  |
|  | M7 | 118 | -6,659.86 | | p = 0.08164 q = 0.85510 | |  |
|  | M8 | 120 | -6,644.48 | | p0 = 0.98074 p = 0.13406 q = 2.69666 | (p1 = 0.01926) w = 1.57044 |  |
| *atp*B | M1 | 118 | -6,126.85 | | p= 0.95457 0.04543 | w= 0.01871 1.00000 |  |
|  | M2 | 120 | -6,126.35 | | p= 0.95535 0.04154 0.00311 | w= 0.01914 1.00000 2.41621 |  |
|  | M7 | 118 | -6,133.38 | | p = 0.06453 q = 0.80739 | |  |
|  | M8 | 120 | -6,123.36 | | p0 = 0.99339 p = 0.07507 q = 1.30171 | (p1 = 0.00661) w = 2.23000 |  |
| *atp*E | M1 | 118 | -1,775.56 | | p= 0.87892 0.12108 | w= 0.06096 1.00000 |  |
|  | M2 | 120 | -1,775.56 | | p= 0.87892 0.12108 0.00000 | w= 0.06096 1.00000 18.23150 |  |
|  | M7 | 118 | -1,775.18 | | p = 0.26566 q = 1.54129 | |  |
|  | M8 | 120 | -1,775.18 | | p0 = 0.99999 p = 0.26567 q = 1.54143 | (p1 = 0.00001) w = 1.00000 |  |
| *atp*F | M1 | 118 | -2,410.99 | | p= 0.85497 0.14503 | w= 0.07988 1.00000 |  |
|  | M2 | 120 | -2,410.99 | | p= 0.85497 0.10847 0.03657 | w= 0.07988 1.00000 1.00000 |  |
|  | M7 | 118 | -2,410.46 | | p = 0.23720 q = 0.95236 | |  |
|  | M8 | 120 | -2,409.21 | | p0 = 0.94042 p = 0.41361 q = 2.58389 | (p1 = 0.05958) w = 1.27187 |  |
| *atp*H | M1 | 118 | -678.88 | | p= 0.98633 0.01367 | w= 0.00392 1.00000 |  |
|  | M2 | 120 | -678.88 | | p= 0.98633 0.01367 0.00000 | w= 0.00392 1.00000 15.27919 |  |
|  | M7 | 118 | -678.46 | | p = 0.01415 q = 0.36464 | |  |
|  | M8 | 120 | -678.46 | | p0 = 0.99999 p = 0.01410 q = 0.36247 | (p1 = 0.00001) w = 4.23414 |  |
| *atp*I | M1 | 118 | -2,808.26 | | p= 0.96608 0.03392 | w= 0.03984 1.00000 |  |
|  | M2 | 120 | -2,808.26 | | p= 0.96608 0.03392 0.00000 | w= 0.03984 1.00000 30.58017 |  |
|  | M7 | 118 | -2,804.05 | | p = 0.12066 q = 1.50263 | |  |
|  | M8 | 120 | -2,802.15 | | p0 = 0.99340 p = 0.15540 q = 2.34222 | (p1 = 0.00660) w = 1.81307 |  |
| *ccs*A | M1 | 118 | -6,231.25 | | p= 0.77722 0.22278 | w= 0.10178 1.00000 |  |
|  | M2 | 120 | -6,216.72 | | p= 0.76466 0.21390 0.02143 | w= 0.10512 1.00000 3.50969 | 194 G 1.000** |
|  | M7 | 118 | -6,224.27 | | p = 0.27060 q = 0.71839 | |  |
|  | M8 | 120 | -6,202.98 | | p0 = 0.96985 p = 0.35849 q = 1.15402 | (p1 = 0.03015) w = 2.81118 | 174 Q 0.996** 194 G 1.000** |
| *cem*A | M1 | 118 | -3,735.10 | | p= 0.75095 0.24905 | w= 0.15970 1.00000 |  |
|  | M2 | 120 | -3,728.94 | | p= 0.74386 0.24557 0.01057 | w= 0.16617 1.00000 4.94010 |  |
|  | M7 | 118 | -3,733.45 | | p = 0.47541 q = 0.91169 | |  |
|  | M8 | 120 | -3,724.89 | | p0 = 0.98852 p = 0.59366 q = 1.23842 | (p1 = 0.01148) w = 4.49423 |  |
| *clp*P | M1 | 118 | -2,082.54 | | p= 0.92788 0.07212 | w= 0.03516 1.00000 |  |
|  | M2 | 120 | -2,082.54 | | p= 0.92788 0.07212 0.00000 | w= 0.03516 1.00000 36.59405 |  |
|  | M7 | 118 | -2,079.67 | | p = 0.11446 q = 1.13245 | |  |
|  | M8 | 120 | -2,079.67 | | p0 = 0.99999 p = 0.11446 q = 1.13255 | (p1 = 0.00001) w = 1.00000 |  |
| *inf*A | M1 | 118 | -389.92 | | p= 0.96459 0.03541 | w= 0.04658 1.00000 |  |
|  | M2 | 120 | -389.60 | | p= 0.96664 0.00000 0.03336 | w= 0.04773 1.00000 1.80853 |  |
|  | M7 | 118 | -391.90 | | p = 0.79304 q = 9.81002 | |  |
|  | M8 | 120 | -389.73 | | p0 = 0.96684 p = 5.14583 q = 99.00000 | (p1 = 0.03316) w = 1.81422 |  |
| *mat*K | M1 | 118 | -5,176.45 | | p= 0.70484 0.29516 | w= 0.15992 1.00000 |  |
|  | M2 | 120 | -5,169.87 | | p= 0.69708 0.26681 0.03611 | w= 0.17308 1.00000 2.73330 |  |
|  | M7 | 118 | -5,180.10 | | p = 0.47931 q = 0.76446 | |  |
|  | M8 | 120 | -5,166.09 | | p0 = 0.91597 p = 0.80106 q = 1.75222 | (p1 = 0.08403) w = 1.97582 |  |
| *ndh*A | M1 | 118 | -5,355.63 | | p= 0.87569 0.12431 | w= 0.04520 1.00000 |  |
|  | M2 | 120 | -5,345.86 | | p= 0.87506 0.11960 0.00534 | w= 0.04615 1.00000 7.02539 | 119 R 0.997** |
|  | M7 | 118 | -5,358.93 | | p = 0.10267 q = 0.51361 | |  |
|  | M8 | 120 | -5,341.24 | | p0 = 0.95019 p = 0.19745 q = 1.76076 | (p1 = 0.04981) w = 2.00242 | 94 Y 0.994** 119 R 0.999** 283 Y 0.992** |
| *ndh*B | M1 | 118 | -2,879.64 | | p= 0.84269 0.15731 | w= 0.00000 1.00000 |  |
|  | M2 | 120 | -2,878.74 | | p= 0.88472 0.00000 0.11528 | w= 0.00000 1.00000 1.61201 |  |
|  | M7 | 118 | -2,882.54 | | p = 0.00500 q = 0.01435 | |  |
|  | M8 | 120 | -2,878.74 | | p0 = 0.88471 p = 0.00500 q = 1.64074 | (p1 = 0.11529) w = 1.61200 |  |
| *ndh*C | M1 | 118 | -1,143.83 | | p= 0.90449 0.09551 | w= 0.03142 1.00000 |  |
|  | M2 | 120 | -1,143.83 | | p= 0.90449 0.09551 0.00000 | w= 0.03142 1.00000 23.90392 |  |
|  | M7 | 118 | -1,143.21 | | p = 0.09399 q = 0.74474 | |  |
|  | M8 | 120 | -1,143.03 | | p0 = 0.99531 p = 0.10176 q = 0.86748 | (p1 = 0.00469) w = 4.52305 |  |
| *ndh*D | M1 | 118 | -8,708.55 | | p= 0.90736 0.09264 | w= 0.07248 1.00000 |  |
|  | M2 | 120 | -8,705.31 | | p= 0.90714 0.09080 0.00206 | w= 0.07319 1.00000 4.57670 |  |
|  | M7 | 118 | -8,694.08 | | p = 0.21276 q = 1.15723 | |  |
|  | M8 | 120 | -8,676.65 | | p0 = 0.97053 p = 0.34559 q = 2.87443 | (p1 = 0.02947) w = 1.55629 |  |
| *ndh*E | M1 | 118 | -1,328.10 | | p= 0.94210 0.05790 | w= 0.02826 1.00000 |  |
|  | M2 | 120 | -1,328.10 | | p= 0.94210 0.05790 0.00000 | w= 0.02826 1.00000 25.84587 |  |
|  | M7 | 118 | -1,331.95 | | p = 0.11059 q = 1.02718 | |  |
|  | M8 | 120 | -1,328.00 | | p0 = 0.95550 p = 0.61103 q = 16.36184 | (p1 = 0.04450) w = 1.10910 |  |
| *ndh*F | M1 | 118 | -8,074.78 | | p= 0.87065 0.12935 | w= 0.03974 1.00000 |  |
|  | M2 | 120 | -8,072.07 | | p= 0.87039 0.11973 0.00988 | w= 0.04067 1.00000 2.80302 |  |
|  | M7 | 118 | -8,056.14 | | p = 0.15154 q = 0.93983 | |  |
|  | M8 | 120 | -8,039.83 | | p0 = 0.97850 p = 0.19261 q = 1.68946 | (p1 = 0.02150) w = 1.98393 |  |
| *ndh*G | M1 | 118 | -2,896.68 | | p= 0.81744 0.18256 | w= 0.05568 1.00000 |  |
|  | M2 | 120 | -2,896.68 | | p= 0.81744 0.18256 0.00000 | w= 0.05568 1.00000 25.64471 |  |
|  | M7 | 118 | -2,890.47 | | p = 0.18727 q = 0.79999 | |  |
|  | M8 | 120 | -2,889.46 | | p0 = 0.95853 p = 0.24198 q = 1.42696 | (p1 = 0.04147) w = 1.43627 |  |
| *ndh*H | M1 | 118 | -5,588.51 | | p= 0.94192 0.05808 | w= 0.02905 1.00000 |  |
|  | M2 | 120 | -5,588.51 | | p= 0.94192 0.05808 0.00000 | w= 0.02905 1.00000 35.35575 |  |
|  | M7 | 118 | -5,572.82 | | p = 0.12046 q = 1.53748 | |  |
|  | M8 | 120 | -5,572.16 | | p0 = 0.98597 p = 0.13972 q = 2.23323 | (p1 = 0.01403) w = 1.00000 |  |
| *ndh*I | M1 | 118 | -2,165.88 | | p= 0.92255 0.07745 | w= 0.02350 1.00000 |  |
|  | M2 | 120 | -2,165.85 | | p= 0.92297 0.07475 0.00228 | w= 0.02375 1.00000 2.40533 |  |
|  | M7 | 118 | -2,161.89 | | p = 0.08530 q = 0.82933 | |  |
|  | M8 | 120 | -2,158.19 | | p0 = 0.97571 p = 0.11587 q = 1.95347 | (p1 = 0.02429) w = 1.62314 |  |
| *ndh*J | M1 | 118 | -1,980.45 | | p= 0.91764 0.08236 | w= 0.02287 1.00000 |  |
|  | M2 | 120 | -1,978.90 | | p= 0.91868 0.07247 0.00885 | w= 0.02399 1.00000 3.51971 |  |
|  | M7 | 118 | -1,982.73 | | p = 0.05868 q = 0.43049 | |  |
|  | M8 | 120 | -1,978.47 | | p0 = 0.97501 p = 0.10687 q = 1.48071 | (p1 = 0.02499) w = 2.18194 |  |
| *ndh*K | M1 | 118 | -2,987.86 | | p= 0.89342 0.10658 | w= 0.03910 1.00000 |  |
|  | M2 | 120 | -2,987.86 | | p= 0.89342 0.07835 0.02823 | w= 0.03910 1.00000 1.00000 |  |
|  | M7 | 118 | -2,986.07 | | p = 0.12538 q = 0.86629 | |  |
|  | M8 | 120 | -2,985.31 | | p0 = 0.92768 p = 0.23978 q = 3.52946 | (p1 = 0.07232) w = 1.00000 |  |
| *pet*A | M1 | 118 | -4,355.32 | | p= 0.94083 0.05917 | w= 0.04785 1.00000 |  |
|  | M2 | 120 | -4,354.23 | | p= 0.94578 0.04232 0.01191 | w= 0.05023 1.00000 2.22567 |  |
|  | M7 | 118 | -4,360.06 | | p = 0.15093 q = 1.17541 | |  |
|  | M8 | 120 | -4,349.79 | | p0 = 0.96886 p = 0.34703 q = 4.80517 | (p1 = 0.03114) w = 1.67732 |  |
| *pet*B | M1 | 118 | -2,451.56 | | p= 0.96587 0.03413 | w= 0.00000 1.00000 |  |
|  | M2 | 120 | -2,451.56 | | p= 0.96587 0.03413 0.00000 | w= 0.00000 1.00000 25.05551 |  |
|  | M7 | 118 | -2,460.78 | | p = 0.01065 q = 0.14599 | |  |
|  | M8 | 120 | -2,451.56 | | p0 = 0.96587 p = 0.00500 q = 81.99042 | (p1 = 0.03413) w = 1.00000 |  |
| *pet*D | M1 | 118 | -1,691.23 | | p= 0.98084 0.01916 | w= 0.00922 1.00000 |  |
|  | M2 | 120 | -1,691.23 | | p= 0.98084 0.01916 0.00000 | w= 0.00922 1.00000 34.77553 |  |
|  | M7 | 118 | -1,699.43 | | p = 0.01160 q = 0.06541 | |  |
|  | M8 | 120 | -1,689.96 | | p0 = 0.98393 p = 0.01518 q = 0.40348 | (p1 = 0.01607) w = 1.00000 |  |
| *pet*G | M1 | 116 | -289.78 | | p= 0.99999 0.00001 | w= 0.00000 1.00000 |  |
|  | M2 | 118 | -289.77 | | p= 1.00000 0.00000 0.00000 | w= 0.00000 1.00000 36.43134 |  |
|  | M7 | 116 | -289.77 | | p = 0.00500 q = 99.00000 | |  |
|  | M8 | 118 | -289.78 | | p0 = 0.99999 p = 0.00500 q = 99.00000 | (p1 = 0.00001) w = 1.00000 |  |
| *pet*L | M1 | 118 | -346.74 | | p= 0.95693 0.04307 | w= 0.08290 1.00000 |  |
|  | M2 | 120 | -346.53 | | p= 0.96555 0.00000 0.03445 | w= 0.08464 1.00000 2.13516 |  |
|  | M7 | 118 | -347.17 | | p = 4.01004 q = 33.73285 | |  |
|  | M8 | 120 | -346.58 | | p0 = 0.96654 p = 9.41784 q = 99.00000 | (p1 = 0.03346) w = 2.14016 |  |
| *pet*N | M1 | 118 | -199.76 | | p= 0.99999 0.00001 | w= 0.06713 1.00000 |  |
|  | M2 | 120 | -199.76 | | p= 1.00000 0.00000 0.00000 | w= 0.06713 1.00000 32.19626 |  |
|  | M7 | 118 | -199.78 | | p = 7.23378 q = 99.00000 | |  |
|  | M8 | 120 | -199.78 | | p0 = 0.99999 p = 7.23353 q = 99.00000 | (p1 = 0.00001) w = 1.00000 |  |
| *psa*A | M1 | 118 | -7,857.09 | | p= 0.97677 0.02323 | w= 0.00981 1.00000 |  |
|  | M2 | 120 | -7,856.87 | | p= 0.97708 0.02204 0.00088 | w= 0.00992 1.00000 3.36982 |  |
|  | M7 | 118 | -7,864.40 | | p = 0.01424 q = 0.25550 | |  |
|  | M8 | 120 | -7,855.47 | | p0 = 0.98418 p = 0.01538 q = 0.37929 | (p1 = 0.01582) w = 1.00000 |  |
| *psa*B | M1 | 118 | -7,508.23 | | p= 0.97766 0.02234 | w= 0.01013 1.00000 |  |
|  | M2 | 120 | -7,508.23 | | p= 0.97766 0.02234 0.00000 | w= 0.01013 1.00000 22.17164 |  |
|  | M7 | 118 | -7,518.39 | | p = 0.01280 q = 0.22106 | |  |
|  | M8 | 120 | -7,500.95 | | p0 = 0.98951 p = 0.00898 q = 0.17726 | (p1 = 0.01049) w = 1.58065 |  |
| *psa*C | M1 | 118 | -837.43 | | p= 0.99999 0.00001 | w= 0.00364 1.00000 |  |
|  | M2 | 120 | -837.43 | | p= 1.00000 0.00000 0.00000 | w= 0.00364 1.00000 67.41973 |  |
|  | M7 | 118 | -837.49 | | p = 0.40407 q = 99.00000 | |  |
|  | M8 | 120 | -837.49 | | p0 = 0.99999 p = 0.40409 q = 99.00000 | (p1 = 0.00001) w = 4.61360 |  |
| *psa*I | M1 | 116 | -467.55 | | p= 0.62606 0.37394 | w= 0.11940 1.00000 |  |
|  | M2 | 118 | -465.23 | | p= 0.61718 0.31790 0.06492 | w= 0.14320 1.00000 5.47501 |  |
|  | M7 | 116 | -468.82 | | p = 0.32414 q = 0.39787 | |  |
|  | M8 | 118 | -465.73 | | p0 = 0.92088 p = 0.53255 q = 0.80408 | (p1 = 0.07912) w = 4.68288 |  |
| *psa*J | M1 | 118 | -478.82 | | p= 0.91867 0.08133 | w= 0.01509 1.00000 |  |
|  | M2 | 120 | -478.82 | | p= 0.91867 0.08133 0.00000 | w= 0.01509 1.00000 24.91181 |  |
|  | M7 | 118 | -476.52 | | p = 0.01619 q = 0.26202 | |  |
|  | M8 | 120 | -476.52 | | p0 = 0.99999 p = 0.01668 q = 0.27021 | (p1 = 0.00001) w = 4.25463 |  |
| *psb*A | M1 | 118 | -3,303.78 | | p= 0.98568 0.01432 | w= 0.00227 1.00000 |  |
|  | M2 | 120 | -3,303.78 | | p= 0.98568 0.01432 0.00000 | w= 0.00227 1.00000 11.38100 |  |
|  | M7 | 118 | -3,308.36 | | p = 0.01357 q = 0.32184 | |  |
|  | M8 | 120 | -3,308.36 | | p0 = 0.99999 p = 0.01351 q = 0.31978 | (p1 = 0.00001) w = 5.11660 |  |
| *psb*B | M1 | 118 | -6,176.81 | | p= 0.97015 0.02985 | w= 0.01214 1.00000 |  |
|  | M2 | 120 | -6,176.81 | | p= 0.97015 0.02985 0.00000 | w= 0.01214 1.00000 34.46464 |  |
|  | M7 | 118 | -6,169.45 | | p = 0.01681 q = 0.31284 | |  |
|  | M8 | 120 | -6,167.85 | | p0 = 0.99336 p = 0.01612 q = 0.32539 | (p1 = 0.00664) w = 1.00000 |  |
| *psb*C | M1 | 118 | -4,955.32 | | p= 0.96664 0.03336 | w= 0.00824 1.00000 |  |
|  | M2 | 120 | -4,955.32 | | p= 0.96664 0.03336 0.00000 | w= 0.00824 1.00000 22.34830 |  |
|  | M7 | 118 | -4,957.07 | | p = 0.01384 q = 0.23452 | |  |
|  | M8 | 120 | -4,951.73 | | p0 = 0.98730 p = 0.01403 q = 0.29685 | (p1 = 0.01270) w = 1.22646 |  |
| *psb*D | M1 | 118 | -3,421.32 | | p= 0.97938 0.02062 | w= 0.00128 1.00000 |  |
|  | M2 | 120 | -3,421.32 | | p= 0.97938 0.02062 0.00000 | w= 0.00128 1.00000 80.53538 |  |
|  | M7 | 118 | -3,435.34 | | p = 0.01130 q = 0.20265 | |  |
|  | M8 | 120 | -3,421.46 | | p0 = 0.97954 p = 0.01837 q = 2.04157 | (p1 = 0.02046) w = 1.00000 |  |
| *psb*E | M1 | 118 | -661.99 | | p= 0.99999 0.00001 | w= 0.00000 1.00000 |  |
|  | M2 | 120 | -661.99 | | p= 1.00000 0.00000 0.00000 | w= 0.00000 1.00000 27.95625 |  |
|  | M7 | 118 | -661.99 | | p = 0.00500 q = 99.00000 | |  |
|  | M8 | 120 | -661.99 | | p0 = 0.99999 p = 0.00500 q = 99.00000 | (p1 = 0.00001) w = 5.58846 |  |
| *psb*F | M1 | 118 | -309.95 | | p= 0.84222 0.15778 | w= 0.00000 1.00000 |  |
|  | M2 | 120 | -309.45 | | p= 0.86437 0.00000 0.13563 | w= 0.00000 1.00000 1.59778 |  |
|  | M7 | 118 | -310.06 | | p = 0.00500 q = 0.03742 | |  |
|  | M8 | 120 | -309.45 | | p0 = 0.86437 p = 0.00500 q = 99.00000 | (p1 = 0.13563) w = 1.59777 |  |
| *psb*H | M1 | 118 | -825.66 | | p= 0.90718 0.09282 | w= 0.02666 1.00000 |  |
|  | M2 | 120 | -825.66 | | p= 0.90718 0.07405 0.01876 | w= 0.02666 1.00000 1.00000 |  |
|  | M7 | 118 | -826.08 | | p = 0.07528 q = 0.56138 | |  |
|  | M8 | 120 | -825.10 | | p0 = 0.96234 p = 0.13999 q = 2.04089 | (p1 = 0.03766) w = 1.37465 |  |
| *psb*I | M1 | 118 | -291.43 | | p= 0.99999 0.00001 | w= 0.01396 1.00000 |  |
|  | M2 | 120 | -291.43 | | p= 1.00000 0.00000 0.00000 | w= 0.01396 1.00000 27.75817 |  |
|  | M7 | 118 | -291.43 | | p = 1.45693 q = 99.00000 | |  |
|  | M8 | 120 | -291.43 | | p0 = 0.99999 p = 1.45677 q = 99.00000 | (p1 = 0.00001) w = 2.08689 |  |
| *psb*J | M1 | 118 | -326.82 | | p= 0.89414 0.10586 | w= 0.02882 1.00000 |  |
|  | M2 | 120 | -326.82 | | p= 0.89414 0.10586 0.00000 | w= 0.02882 1.00000 14.78054 |  |
|  | M7 | 118 | -327.03 | | p = 0.02988 q = 0.16655 | |  |
|  | M8 | 120 | -326.71 | | p0 = 0.91462 p = 3.79023 q = 99.00000 | (p1 = 0.08538) w = 1.34262 |  |
| *psb*K | M1 | 118 | -661.48 | | p= 0.81829 0.18171 | w= 0.03558 1.00000 |  |
|  | M2 | 120 | -661.48 | | p= 0.81829 0.18171 0.00000 | w= 0.03558 1.00000 17.58666 |  |
|  | M7 | 118 | -661.42 | | p = 0.12031 q = 0.55063 | |  |
|  | M8 | 120 | -661.40 | | p0 = 0.88138 p = 0.17884 q = 1.94067 | (p1 = 0.11862) w = 1.00000 |  |
| *psb*L | M1 | 118 | -204.27 | | p= 0.99999 0.00001 | w= 0.07968 1.00000 |  |
|  | M2 | 120 | -204.27 | | p= 1.00000 0.00000 0.00000 | w= 0.07969 1.00000 1.00000 |  |
|  | M7 | 118 | -204.27 | | p = 8.65248 q = 99.00000 | |  |
|  | M8 | 120 | -204.27 | | p0 = 0.99999 p = 8.65247 q = 99.00000 | (p1 = 0.00001) w = 48.54438 |  |
| *psb*M | M1 | 118 | -313.44 | | p= 0.90028 0.09972 | w= 0.00000 1.00000 |  |
|  | M2 | 120 | -311.79 | | p= 0.90181 0.06308 0.03511 | w= 0.00000 1.00000 3.35977 |  |
|  | M7 | 118 | -313.53 | | p = 0.00500 q = 0.03803 | |  |
|  | M8 | 120 | -312.25 | | p0 = 0.90721 p = 0.00500 q = 99.00000 | (p1 = 0.09279) w = 1.94181 |  |
| *psb*N | M1 | 118 | -315.34 | | p= 0.99999 0.00001 | w= 0.04347 1.00000 |  |
|  | M2 | 120 | -315.34 | | p= 1.00000 0.00000 0.00000 | w= 0.04347 1.00000 1.00000 |  |
|  | M7 | 118 | -315.36 | | p = 4.58259 q = 99.00000 | |  |
|  | M8 | 120 | -315.36 | | p0 = 0.99999 p = 4.58243 q = 99.00000 | (p1 = 0.00001) w = 1.00000 |  |
| *psb*T | M1 | 118 | -340.49 | | p= 0.92659 0.07341 | w= 0.02793 1.00000 |  |
|  | M2 | 120 | -340.49 | | p= 0.92659 0.07341 0.00000 | w= 0.02793 1.00000 28.57707 |  |
|  | M7 | 118 | -341.55 | | p = 0.06475 q = 0.45494 | |  |
|  | M8 | 120 | -340.34 | | p0 = 0.93532 p = 3.28212 q = 99.00000 | (p1 = 0.06468) w = 1.35276 |  |
| *psb*Z | M1 | 118 | -562.76 | | p= 0.97090 0.02910 | w= 0.04879 1.00000 |  |
|  | M2 | 120 | -563.22 | | p= 0.97088 0.02912 0.00000 | w= 0.04887 1.00000 28.49120 |  |
|  | M7 | 118 | -569.84 | | p = 0.01157 q = 0.02762 | |  |
|  | M8 | 120 | -562.02 | | p0 = 0.99999 p = 0.23322 q = 2.95962 | (p1 = 0.00001) w = 1.00000 |  |
| *rbc*L | M1 | 118 | -5,475.39 | | p= 0.92482 0.07518 | w= 0.00517 1.00000 |  |
|  | M2 | 120 | -5,459.93 | | p= 0.92505 0.06326 0.01169 | w= 0.00554 1.00000 3.89301 | 32 L 0.998** 279 T 1.000** 449 A 0.990** |
|  | M7 | 118 | -5,480.68 | | p = 0.01243 q = 0.06881 | |  |
|  | M8 | 120 | -5,463.97 | | p0 = 0.96520 p = 0.01290 q = 0.22223 | (p1 = 0.03480) w = 2.16044 | 32 L 1.000** 225 L 0.991** 279 T 1.000** 439 R 0.997** 443 D 0.993** 449 A 0.999** 470 E 0.992** |
| *rpl*14 | M1 | 118 | -1,373.31 | | p= 0.92214 0.07786 | w= 0.02867 1.00000 |  |
|  | M2 | 120 | -1,373.31 | | p= 0.92214 0.07786 0.00000 | w= 0.02867 1.00000 32.97520 |  |
|  | M7 | 118 | -1,374.21 | | p = 0.06892 q = 0.52328 | |  |
|  | M8 | 120 | -1,372.82 | | p0 = 0.94321 p = 0.26406 q = 5.83212 | (p1 = 0.05679) w = 1.23761 |  |
| *rpl*16 | M1 | 118 | -1,917.37 | | p= 0.96665 0.03335 | w= 0.03996 1.00000 |  |
|  | M2 | 120 | -1,917.37 | | p= 0.96665 0.02542 0.00792 | w= 0.03996 1.00000 1.00000 |  |
|  | M7 | 118 | -1,912.01 | | p = 0.16924 q = 2.28132 | |  |
|  | M8 | 120 | -1,912.01 | | p0 = 0.99999 p = 0.16920 q = 2.28044 | (p1 = 0.00001) w = 4.04622 |  |
| *rpl*2 | M1 | 118 | -1,960.72 | | p= 0.84291 0.15709 | w= 0.03574 1.00000 |  |
|  | M2 | 120 | -1,960.72 | | p= 0.84291 0.15709 0.00000 | w= 0.03574 1.00000 41.02687 |  |
|  | M7 | 118 | -1,960.48 | | p = 0.08012 q = 0.36725 | |  |
|  | M8 | 120 | -1,960.48 | | p0 = 0.99999 p = 0.08013 q = 0.36730 | (p1 = 0.00001) w = 1.00000 |  |
| *rpl*20 | M1 | 118 | -1,935.76 | | p= 0.83599 0.16401 | w= 0.10289 1.00000 |  |
|  | M2 | 120 | -1,935.76 | | p= 0.83599 0.16401 0.00000 | w= 0.10289 1.00000 24.21837 |  |
|  | M7 | 118 | -1,935.25 | | p = 0.32570 q = 1.07565 | |  |
|  | M8 | 120 | -1,932.78 | | p0 = 0.98309 p = 0.41929 q = 1.61969 | (p1 = 0.01691) w = 2.63689 |  |
| *rpl*22 | M1 | 118 | -5,826.70 | | p= 0.56858 0.43142 | w= 0.06469 1.00000 |  |
|  | M2 | 120 | -5,807.29 | | p= 0.54828 0.41304 0.03868 | w= 0.06085 1.00000 3.08779 | 6 W 0.999** 145 W 0.998** 176 R 0.999** |
|  | M7 | 118 | -5,797.83 | | p = 0.24184 q = 0.44786 | |  |
|  | M8 | 120 | -5,783.07 | | p0 = 0.96271 p = 0.25577 q = 0.50694 | (p1 = 0.03729) w = 2.47991 | 6 W 0.998** 145 W 0.998** 176 R 0.999** |
| *rpl*23 | M1 | 116 | -119.51 | | p= 0.23612 0.76388 | w= 0.00000 1.00000 |  |
|  | M2 | 118 | -119.51 | | p= 0.23612 0.58563 0.17825 | w= 0.00000 1.00000 1.00000 |  |
|  | M7 | 116 | -119.51 | | p = 0.03359 q = 0.01402 | |  |
|  | M8 | 118 | -119.51 | | p0 = 0.99999 p = 0.03194 q = 0.01338 | (p1 = 0.00001) w = 4.99411 |  |
| *rpl*32 | M1 | 118 | -852.67 | | p= 0.94745 0.05255 | w= 0.07370 1.00000 |  |
|  | M2 | 120 | -852.43 | | p= 0.95548 0.00000 0.04452 | w= 0.07749 1.00000 1.35152 |  |
|  | M7 | 118 | -851.28 | | p = 0.26193 q = 1.61154 | |  |
|  | M8 | 120 | -848.76 | | p0 = 0.95907 p = 0.50327 q = 5.43178 | (p1 = 0.04093) w = 1.38628 |  |
| *rpl*33 | M1 | 118 | -865.51 | | p= 0.89428 0.10572 | w= 0.07054 1.00000 |  |
|  | M2 | 120 | -865.51 | | p= 0.89428 0.10572 0.00000 | w= 0.07054 1.00000 25.80414 |  |
|  | M7 | 118 | -865.24 | | p = 0.25280 q = 1.44193 | |  |
|  | M8 | 120 | -865.24 | | p0 = 0.99999 p = 0.25280 q = 1.44193 | (p1 = 0.00001) w = 5.14626 |  |
| *rpl*36 | M1 | 118 | -368.12 | | p= 0.99999 0.00001 | w= 0.02664 1.00000 |  |
|  | M2 | 120 | -368.12 | | p= 1.00000 0.00000 0.00000 | w= 0.02664 1.00000 60.89262 |  |
|  | M7 | 118 | -366.44 | | p = 0.01383 q = 0.24857 | |  |
|  | M8 | 120 | -366.44 | | p0 = 0.99999 p = 0.01382 q = 0.24840 | (p1 = 0.00001) w = 5.12093 |  |
| *rpo*A | M1 | 118 | -5,462.90 | | p= 0.85221 0.14779 | w= 0.09612 1.00000 |  |
|  | M2 | 120 | -5,458.62 | | p= 0.85412 0.12679 0.01909 | w= 0.10171 1.00000 2.69365 |  |
|  | M7 | 118 | -5,474.29 | | p = 0.29753 q = 0.98074 | |  |
|  | M8 | 120 | -5,457.64 | | p0 = 0.91936 p = 0.74377 q = 4.43118 | (p1 = 0.08064) w = 1.55478 |  |
| *rpo*B | M1 | 118 | -6,675.55 | | p= 0.88697 0.11303 | w= 0.04119 1.00000 |  |
|  | M2 | 120 | -6,674.57 | | p= 0.89001 0.10095 0.00904 | w= 0.04314 1.00000 2.50145 |  |
|  | M7 | 118 | -6,673.73 | | p = 0.11002 q = 0.65297 | |  |
|  | M8 | 120 | -6,664.24 | | p0 = 0.96986 p = 0.18817 q = 1.75496 | (p1 = 0.03014) w = 1.97253 |  |
| *rpo*C1 | M1 | 118 | -8,103.39 | | p= 0.90670 0.09330 | w= 0.03918 1.00000 |  |
|  | M2 | 120 | -8,103.39 | | p= 0.90670 0.09330 0.00000 | w= 0.03918 1.00000 26.27029 |  |
|  | M7 | 118 | -8,099.14 | | p = 0.12651 q = 0.96402 | |  |
|  | M8 | 120 | -8,096.04 | | p0 = 0.95027 p = 0.20665 q = 2.75682 | (p1 = 0.04973) w = 1.00000 |  |
| *rpo*C2 | M1 | 116 | -21,419.35 | | p= 0.80834 0.19166 | w= 0.09035 1.00000 |  |
|  | M2 | 118 | -21,412.65 | | p= 0.80802 0.18415 0.00783 | w= 0.09252 1.00000 3.18222 |  |
|  | M7 | 116 | -21,414.34 | | p = 0.25640 q = 0.80237 | |  |
|  | M8 | 118 | -21,390.97 | | p0 = 0.95496 p = 0.37372 q = 1.57565 | (p1 = 0.04504) w = 1.77203 |  |
| *rps*11 | M1 | 118 | -2,195.47 | | p= 0.90519 0.09481 | w= 0.02982 1.00000 |  |
|  | M2 | 120 | -2,195.47 | | p= 0.90519 0.04547 0.04935 | w= 0.02982 1.00000 1.00000 |  |
|  | M7 | 118 | -2,186.66 | | p = 0.14238 q = 1.50204 | |  |
|  | M8 | 120 | -2,186.66 | | p0 = 0.99999 p = 0.14239 q = 1.50225 | (p1 = 0.00001) w = 1.00000 |  |
| *rps*12 | M1 | 118 | -881.04 | | p= 0.80594 0.19406 | w= 0.01096 1.00000 |  |
|  | M2 | 120 | -870.82 | | p= 0.78051 0.20945 0.01004 | w= 0.00417 1.00000 12.25494 | 100 Q 1.000** |
|  | M7 | 118 | -881.34 | | p = 0.00509 q = 0.01554 | |  |
|  | M8 | 120 | -870.99 | | p0 = 0.99002 p = 0.01190 q = 0.04448 | (p1 = 0.00998) w = 12.53978 | 100 Q 1.000** |
| *rps*14 | M1 | 118 | -1,263.54 | | p= 0.86604 0.13396 | w= 0.04208 1.00000 |  |
|  | M2 | 120 | -1,262.07 | | p= 0.86594 0.11669 0.01737 | w= 0.04524 1.00000 2.93857 |  |
|  | M7 | 118 | -1,263.72 | | p = 0.08539 q = 0.42252 | |  |
|  | M8 | 120 | -1,261.07 | | p0 = 0.96799 p = 0.14349 q = 0.98988 | (p1 = 0.03201) w = 2.33196 |  |
| *rps*15 | M1 | 118 | -1,579.12 | | p= 0.83812 0.16188 | w= 0.08452 1.00000 |  |
|  | M2 | 120 | -1,576.74 | | p= 0.84559 0.09809 0.05632 | w= 0.09231 1.00000 1.63980 |  |
|  | M7 | 118 | -1,580.00 | | p = 0.23251 q = 0.73007 | |  |
|  | M8 | 120 | -1,576.07 | | p0 = 0.88709 p = 0.69518 q = 4.97735 | (p1 = 0.11291) w = 1.45192 |  |
| *rps*16 | M1 | 118 | -889.08 | | p= 0.89483 0.10517 | w= 0.06707 1.00000 |  |
|  | M2 | 120 | -889.08 | | p= 0.89483 0.10517 0.00000 | w= 0.06707 1.00000 33.90824 |  |
|  | M7 | 118 | -891.18 | | p = 0.23000 q = 1.17205 | |  |
|  | M8 | 120 | -889.12 | | p0 = 0.89592 p = 7.27566 q = 99.00000 | (p1 = 0.10408) w = 1.00000 |  |
| *rps*18 | M1 | 118 | -1,180.93 | | p= 0.87015 0.12985 | w= 0.03201 1.00000 |  |
|  | M2 | 120 | -1,180.93 | | p= 0.87015 0.12985 0.00000 | w= 0.03201 1.00000 23.81915 |  |
|  | M7 | 118 | -1,178.08 | | p = 0.13163 q = 0.96132 | |  |
|  | M8 | 120 | -1,176.98 | | p0 = 0.98003 p = 0.17448 q = 1.73231 | (p1 = 0.01997) w = 2.38334 |  |
| *rps*19 | M1 | 118 | -1,149.18 | | p= 0.95471 0.04529 | w= 0.07114 1.00000 |  |
|  | M2 | 120 | -1,149.18 | | p= 0.95471 0.04176 0.00354 | w= 0.07114 1.00000 1.00000 |  |
|  | M7 | 118 | -1,143.23 | | p = 0.28523 q = 2.48141 | |  |
|  | M8 | 120 | -1,142.60 | | p0 = 0.98608 p = 0.33178 q = 3.36441 | (p1 = 0.01392) w = 1.93004 |  |
| *rps*2 | M1 | 118 | -2,730.02 | | p= 0.90905 0.09095 | w= 0.03161 1.00000 |  |
|  | M2 | 120 | -2,730.02 | | p= 0.90905 0.09095 0.00000 | w= 0.03161 1.00000 32.12378 |  |
|  | M7 | 118 | -2,729.84 | | p = 0.10041 q = 0.82492 | |  |
|  | M8 | 120 | -2,729.84 | | p0 = 0.99999 p = 0.10041 q = 0.82500 | (p1 = 0.00001) w = 1.00000 |  |
| *rps*3 | M1 | 118 | -3,494.41 | | p= 0.93060 0.06940 | w= 0.04707 1.00000 |  |
|  | M2 | 120 | -3,494.41 | | p= 0.93060 0.06940 0.00000 | w= 0.04707 1.00000 6.15066 |  |
|  | M7 | 118 | -3,487.20 | | p = 0.19541 q = 1.66944 | |  |
|  | M8 | 120 | -3,485.31 | | p0 = 0.99033 p = 0.22788 q = 2.29654 | (p1 = 0.00967) w = 1.65124 |  |
| *rps*4 | M1 | 118 | -2,670.25 | | p= 0.87063 0.12937 | w= 0.05158 1.00000 |  |
|  | M2 | 120 | -2,669.55 | | p= 0.87140 0.12359 0.00501 | w= 0.05278 1.00000 3.62716 |  |
|  | M7 | 118 | -2,672.89 | | p = 0.15710 q = 0.77913 | |  |
|  | M8 | 120 | -2,669.16 | | p0 = 0.94096 p = 0.34601 q = 3.20040 | (p1 = 0.05904) w = 1.47529 |  |
| *rps*7 | M1 | 118 | -907.26 | | p= 0.99999 0.00001 | w= 0.07123 1.00000 |  |
|  | M2 | 120 | -907.26 | | p= 1.00000 0.00000 0.00000 | w= 0.07124 1.00000 10.43335 |  |
|  | M7 | 118 | -907.29 | | p = 7.67563 q = 99.00000 | |  |
|  | M8 | 120 | -907.29 | | p0 = 0.99999 p = 7.67569 q = 99.00000 | (p1 = 0.00001) w = 4.04715 |  |
| *rps*8 | M1 | 118 | -2,072.64 | | p= 0.85933 0.14067 | w= 0.09213 1.00000 |  |
|  | M2 | 120 | -2,070.75 | | p= 0.85995 0.12840 0.01164 | w= 0.09624 1.00000 2.85956 |  |
|  | M7 | 118 | -2,073.31 | | p = 0.33299 q = 1.26635 | |  |
|  | M8 | 120 | -2,068.53 | | p0 = 0.97491 p = 0.49624 q = 2.43759 | (p1 = 0.02509) w = 2.31507 |  |
| *ycf*1 | M1 | 116 | -24,313.75 | | p= 0.62142 0.37858 | w= 0.13131 1.00000 |  |
|  | M2 | 118 | -24,206.11 | | p= 0.57616 0.35699 0.06685 | w= 0.13655 1.00000 3.04216 | 413 T 1.000** 444 R 0.999** 522 P 1.000** 529 F 1.000** 547 R 0.992** 551 K 1.000** 682 H 0.999** 694 N 1.000** 700 S 0.999** 706 I 0.999** 717 L 0.995** 776 I 1.000** 828 W 1.000** 844 E 1.000** 966 S 0.999** |
|  | M7 | 116 | -24,313.76 | | p = 0.33943 q = 0.46523 | |  |
|  | M8 | 118 | -24,187.96 | | p0 = 0.90919 p = 0.42333 q = 0.66174 | (p1 = 0.09081) w = 2.51015 | 413 T 1.000** 444 R 0.999** 522 P 1.000** 529 F 1.000** 547 R 0.994** 551 K 1.000** 682 H 0.997** 694 N 1.000** 700 S 0.997** 706 I 0.999** 717 L 0.993** 776 I 0.999** 828 W 0.999** 844 E 1.000** 966 S 0.999** |
| *ycf*2 | M1 | 118 | -16,368.48 | | p= 0.36841 0.63159 | w= 0.01151 1.00000 |  |
|  | M2 | 120 | -16,256.44 | | p= 0.27423 0.66289 0.06288 | w= 0.00000 1.00000 6.86787 | 124 G 0.999** 223 S 0.999** 300 R 0.999** 307 S 0.991** 308 R 0.999** 318 T 0.994** 340 I 0.998** 411 L 1.000** 847 L 0.995** 1030 A 0.995** 1151 S 0.995** 1429 A 0.999** 1511 Y 0.999** 1513 K 1.000** 1516 I 0.995** 1525 G 0.999** |
|  | M7 | 118 | -16,368.54 | | p = 0.03029 q = 0.01579 | |  |
|  | M8 | 120 | -16,252.26 | | p0 = 0.93604 p = 0.01534 q = 0.00500 | (p1 = 0.06396) w = 7.14059 | 124 G 1.000** 223 S 0.999** 300 R 1.000** 307 S 0.995** 308 R 0.999** 318 T 0.997** 340 I 0.999** 411 L 1.000** 520 S 0.994** 713 Q 0.991** 847 L 0.997** 1030 A 0.997** 1151 S 0.997** 1429 A 1.000** 1511 Y 0.999** 1513 K 1.000** 1516 I 0.998** 1525 G 0.999** |
| *ycf*3 | M1 | 118 | -1,567.20 | | p= 0.96357 0.03643 | w= 0.00986 1.00000 |  |
|  | M2 | 120 | -1,567.20 | | p= 0.96357 0.03643 0.00000 | w= 0.00986 1.00000 31.38931 |  |
|  | M7 | 118 | -1,570.79 | | p = 0.01426 q = 0.21114 | |  |
|  | M8 | 120 | -1,566.33 | | p0 = 0.97200 p = 0.06056 q = 2.95904 | (p1 = 0.02800) w = 1.40384 |  |
| *ycf*4 | M1 | 118 | -2,639.55 | | p= 0.89317 0.10683 | w= 0.05550 1.00000 |  |
|  | M2 | 120 | -2,639.55 | | p= 0.89317 0.10683 0.00000 | w= 0.05550 1.00000 56.39879 |  |
|  | M7 | 118 | -2,642.49 | | p = 0.21738 q = 1.29712 | |  |
|  | M8 | 120 | -2,639.40 | | p0 = 0.90071 p = 1.66289 q = 25.66161 | (p1 = 0.09929) w = 1.00000 |  |

**Table S4** Results of maximum likelihood parameters and test hypotheses for 79 de-redundant PCGs with evidence of positively selected sites based on the branch-site models.

| **Gene** | **Model** | **np** | **lnL** | **Estimates of parameters** | | | | | **LRT P-value** | **Positive sites** |
| --- | --- | --- | --- | --- | --- | --- | --- | --- | --- | --- |
| *acc*D | Model A | 120 | -7326.331398 | Site class | 0 | 1 | 2a | 2b | 1.93E-08 | 58 Q 0.997** 291 P 0.999** 58 Q 0.997** 291 P 0.999** |
|  |  |  |  | proportion | 0.7252 | 0.25056 | 0.01802 | 0.00623 |  |  |
|  |  |  |  | background w | 0.09374 | 1 | 0.09374 | 1 |  |  |
|  |  |  |  | foreground w | 0.09374 | 1 | 4.63372 | 4.63372 |  |  |
|  | Model null | 119 | -7342.112254 |  |  |  |  |  |  |  |
| *ccs*A | Model A | 120 | -6225.56339 | Site class | 0 | 1 | 2a | 2b | 2.33E-02 |  |
|  |  |  |  | proportion | 0.76282 | 0.22586 | 0.00873 | 0.00259 |  |  |
|  |  |  |  | background w | 0.10272 | 1 | 0.10272 | 1 |  |  |
|  |  |  |  | foreground w | 0.10272 | 1 | 4.51254 | 4.51254 |  |  |
|  | Model null | 119 | -6230.196489 |  |  |  |  |  |  |  |
| *ndh*A | Model A | 120 | -5347.190223 | Site class | 0 | 1 | 2a | 2b | 5.09E-05 | 119 R 0.999** 119 R 0.998** |
|  |  |  |  | proportion | 0.87229 | 0.11885 | 0.0078 | 0.00106 |  |  |
|  |  |  |  | background w | 0.04683 | 1 | 0.04683 | 1 |  |  |
|  |  |  |  | foreground w | 0.04683 | 1 | 4.88008 | 4.88008 |  |  |
|  | Model null | 119 | -5355.397253 |  |  |  |  |  |  |  |
| *rbc*L | Model A | 120 | -5463.358406 | Site class | 0 | 1 | 2a | 2b | 9.37E-07 | 279 T 0.997** 449 A 0.999** 279 T 0.993** 449 A 1.000** |
|  |  |  |  | proportion | 0.9206 | 0.07318 | 0.00576 | 0.00046 |  |  |
|  |  |  |  | background w | 0.00531 | 1 | 0.00531 | 1 |  |  |
|  |  |  |  | foreground w | 0.00531 | 1 | 6.25127 | 6.25127 |  |  |
|  | Model null | 119 | -5475.385373 |  |  |  |  |  |  |  |
| *rpl*22 | Model A | 120 | -5811.409359 | Site class | 0 | 1 | 2a | 2b | 4.93E-04 | 6 W 1.000** 145 W 0.999** 6 W 1.000** 145 W 0.999** |
|  |  |  |  | proportion | 0.55095 | 0.43124 | 0.00999 | 0.00782 |  |  |
|  |  |  |  | background w | 0.06189 | 1 | 0.06189 | 1 |  |  |
|  |  |  |  | foreground w | 0.06189 | 1 | 4.85622 | 4.85622 |  |  |
|  | Model null | 119 | -5817.480696 |  |  |  |  |  |  |  |
| *rps*12 | Model A | 120 | -876.304218 | Site class | 0 | 1 | 2a | 2b | 4.42E-02 | 100 Q 0.991** |
|  |  |  |  | proportion | 0.8377 | 0.06307 | 0.09229 | 0.00695 |  |  |
|  |  |  |  | background w | 0.02919 | 1 | 0.02919 | 1 |  |  |
|  |  |  |  | foreground w | 0.02919 | 1 | 3.21323 | 3.21323 |  |  |
|  | Model null | 119 | -878.329369 |  |  |  |  |  |  |  |
| *ycf*1 | Model A | 118 | -24254.72497 | Site class | 0 | 1 | 2a | 2b | 0 | 529 F 1.000** 536 E 0.994** 551 K 1.000** 694 N 0.998** 717 L 0.992** 776 I 0.991** 844 E 0.999** 966 S 0.997** 529 F 1.000** 536 E 0.995** 551 K 1.000** 694 N 0.998** 717 L 0.992** 776 I 0.991** 844 E 0.999** 966 S 0.997** |
|  |  |  |  | proportion | 0.57769 | 0.38112 | 0.02482 | 0.01637 |  |  |
|  |  |  |  | background w | 0.13338 | 1 | 0.13338 | 1 |  |  |
|  |  |  |  | foreground w | 0.13338 | 1 | 3.48809 | 3.48809 |  |  |
|  | Model null | 117 | -24312.26555 |  |  |  |  |  |  |  |
| *ycf*2 | Model A | 120 | -16305.50933 | Site class | 0 | 1 | 2a | 2b | 0 | 223 S 0.992** 318 T 0.996** 340 I 0.993** 411 L 0.994** 847 L 0.996** 1030 A 0.996** 1429 A 0.999** 1513 K 1.000** 223 S 0.992** 318 T 0.996** 340 I 0.994** 411 L 0.994** 847 L 0.996** 1030 A 0.996** 1429 A 0.999** 1513 K 1.000** |
|  |  |  |  | proportion | 0.29456 | 0.62413 | 0.02607 | 0.05524 |  |  |
|  |  |  |  | background w | 0 | 1 | 0 | 1 |  |  |
|  |  |  |  | foreground w | 0 | 1 | 5.67411 | 5.67411 |  |  |
|  | Model null | 119 | -16368.47268 |  |  |  |  |  |  |  |

**Table S5** Molecular dating results derived from the BEAST analysis.

| **Clade/lineage** | **Stem: (age interval) (Ma)** | **Crown: (age interval) (Ma)** |
| --- | --- | --- |
| Amaranthaceae | 69.80 (68.58-71.01) | 62.55 (61.66-63.45) |
| Chenopodioideae | 62.55 (61.66-63.45) | 61.27 (60.35-62.23) |
| Amaranthaceae *s.s.* | 62.55 (61.66-63.45) | 51.08 (50.02-52.18) |
| Clade I | 61.27 (60.35-62.23) | 56.42 (55.35-57.49) |
| Clade II | 61.27 (60.35-62.23) | 48.88 (47.88-49.85) |
| *Agriophyllum*+*Corispermum* | 51.78 (50.47-52.84) | 28.71 (27.53-29.91) |
| Chenopodioideae+Corispermoideae | 56.42 (55.35-57.49) | 51.78 (50.74-52.84) |
| Atripliceae+Chenopodieae | 51.78 (50.47-52.84) | 46.96 (45.96-47.97) |
| *Atriplex*+*Chenopodium* | 29.91 (29.29-30.55) | 25.01 (24.39-25.65) |
| *Atriplex* | 16.95 (16.40-17.51) | 5.01 (4.61-5.4) |
| Suaedoideae+Salicornioideae | 48.88 (47.88-49.85) | 36.50 (35.67-37.35) |
| Salsoloideae | 48.88 (47.88-49.85) | 34.39 (33.39-35.41) |
| Suaedoideae | 36.50 (35.67-37.35) | 32.35 (31.47-33.23) |
| Salicornioideae | 36.50 (35.67-37.35) | 23.34 (23.30-23.46) |
| *Suaeda* | 32.35 (31.47-33.23) | 27.10 (26.27-27.97) |
| *Salicornia* | 23.34 (23.30-23.46) | 3.00 (2.65-3.37) |
| *Kalidium* | 23.34 (23.30-23.46) | 4.48 (3.94-5.05) |
| *Haloxylon* | 7.55 (7.02-8.08) | 0.34 (0.21-0.45) |
| Achyrantheae+Aerveae+Gomphreneae | 51.08 (50.02-52.18) | 27.68 (26.74-28.62) |
| Achyrantheae+Aerveae | 27.68 (26.74-28.62) | 23.77 (22.86-24.66) |
| Achyrantheae | 23.77 (22.86-24.66) | 8.29 (7.64-8.94) |
| *Achyranthes* | 8.29 (7.64-8.94) | 0.65 (0.49-0.81) |
| Amarantheae+Celosieae | 51.08 (50.02-52.18) | 41.23 (40.12-42.36) |
| Amarantheae | 41.23 (40.12-42.36) | 4.44 (4.05-4.84) |
| Celosieae | 41.23 (40.12-42.36) | 36.67 (35.51-37.83) |

**Table S6** Statistics of the three main topologies present in gene trees.

| **Topology** | **((C1, Ama *s.s.*), C2)** | **((C1, C2), Ama *s.s.*)** | **(C2, Ama *s.s.*), C1)** |
| --- | --- | --- | --- |
| **Genes** | *acc*D, *atp*A, *atp*F, *atp*I, *inf*A, *ndh*A, *ndh*K, *pet*L, *psb*E, *psb*K, *rbc*L, *rpl*16, *rpl*32, *rpo*A, *rpo*C2, *rps*16, *rps*3, *ycf*1, *ycf*4 | *atp*B, *atp*H, *clp*P, *mat*K, *ndh*B, *ndh*B_copy, *ndh*D, *ndh*E, *ndh*H, *ndh*J, *pet*B, *pet*D, *pet*N, *psa*A, *psa*J, *psb*B, *psb*C, *psb*D, *psb*F, *psb*H, *psb*J, *psb*L, *psb*M, *psb*T, *psb*Z, *rpl*22, *rpl*2, *rpl*2_copy, *rpl*33, *rps*14, *rps*15, *ycf*2, *ycf*2_copy, *ycf*3 | *atp*E, *ccs*A, *cem*A, *ndh*C, *ndh*F, *ndh*G, *ndh*I, *pet*A, *psa*B, *psa*C, *psb*A, *psb*I, *rpl*14, *rpl*20, *rpl*36, *rpo*B, *rpo*C1, *rps*12, *rps*12_copy, *rps*18, *rps*2, *rps*4, *rps*7, *rps*7_copy, *rps*8 |
| **Total** | 19 | 34 | 25 |


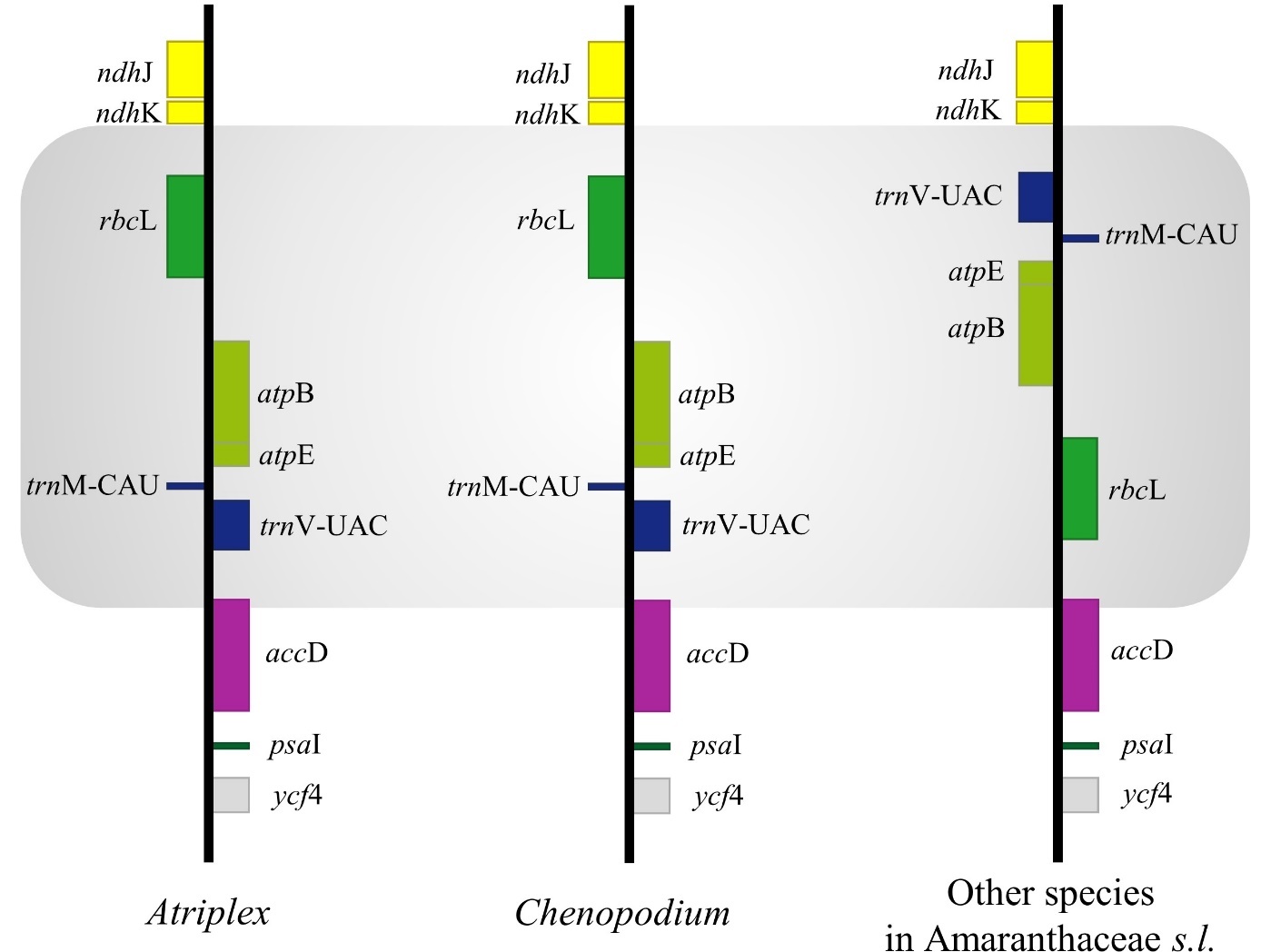


**Figure S1** The plastome structure comparison of *Atriplex*, *Chenopodium*, and remaining Amaranthaceae *s.l.* in this study. The gray region shows that *Atriplex* and *Chenopodium* exist a reverse complement fragment of plastome (*rbc*L-*atp*B-*atp*E-*trn*M^CAU^-*trn*V^UAC^), compared with that of other Amaranthaceae *s.l.*


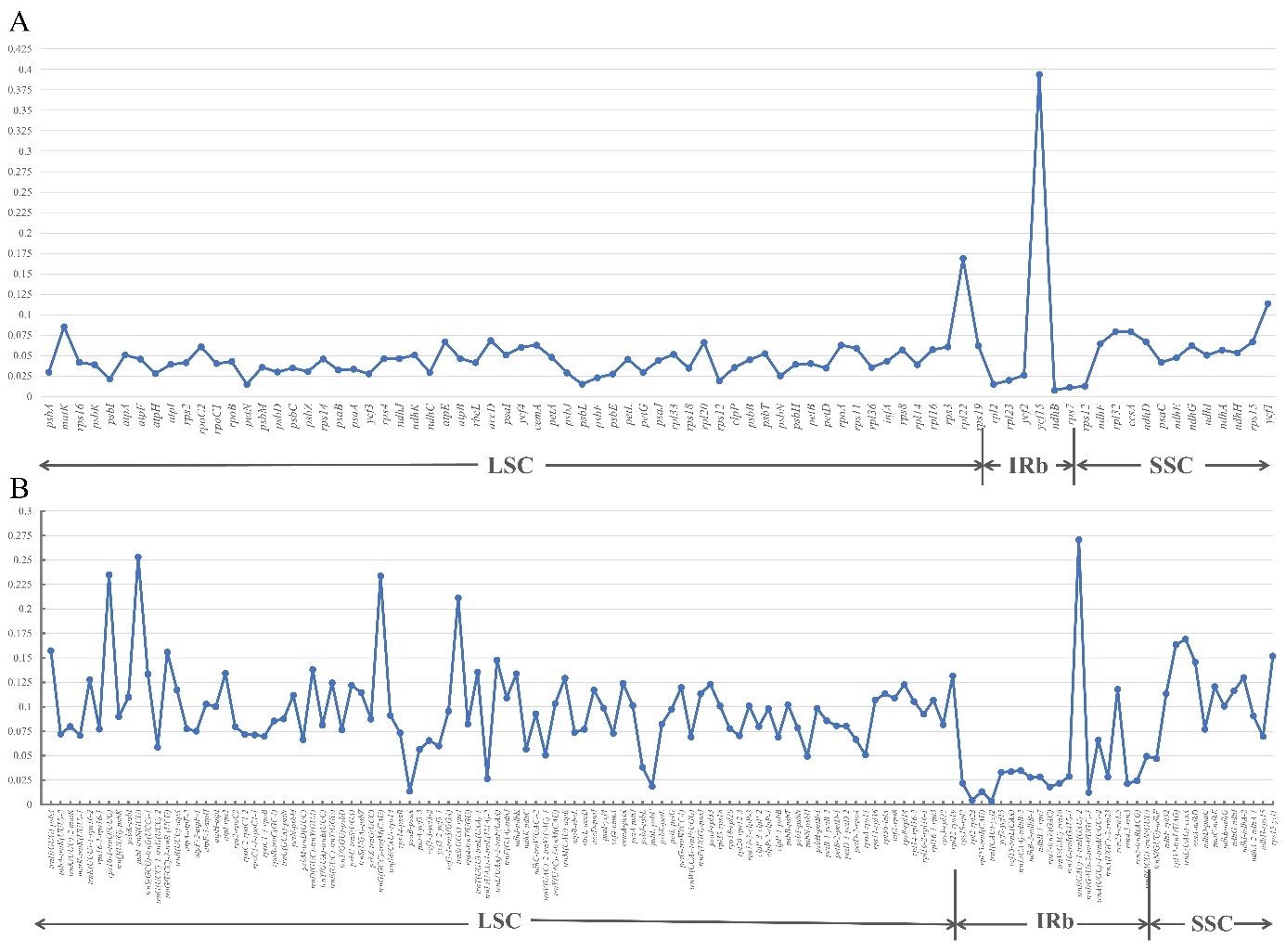


**Figure S2** Comparison of the Pi value based on DnaSP among 59 Amaranthaceae *s.l.* individuals. (A) 79 de-redundant protein-coding regions. (B) 127 intergenic spacer regions.


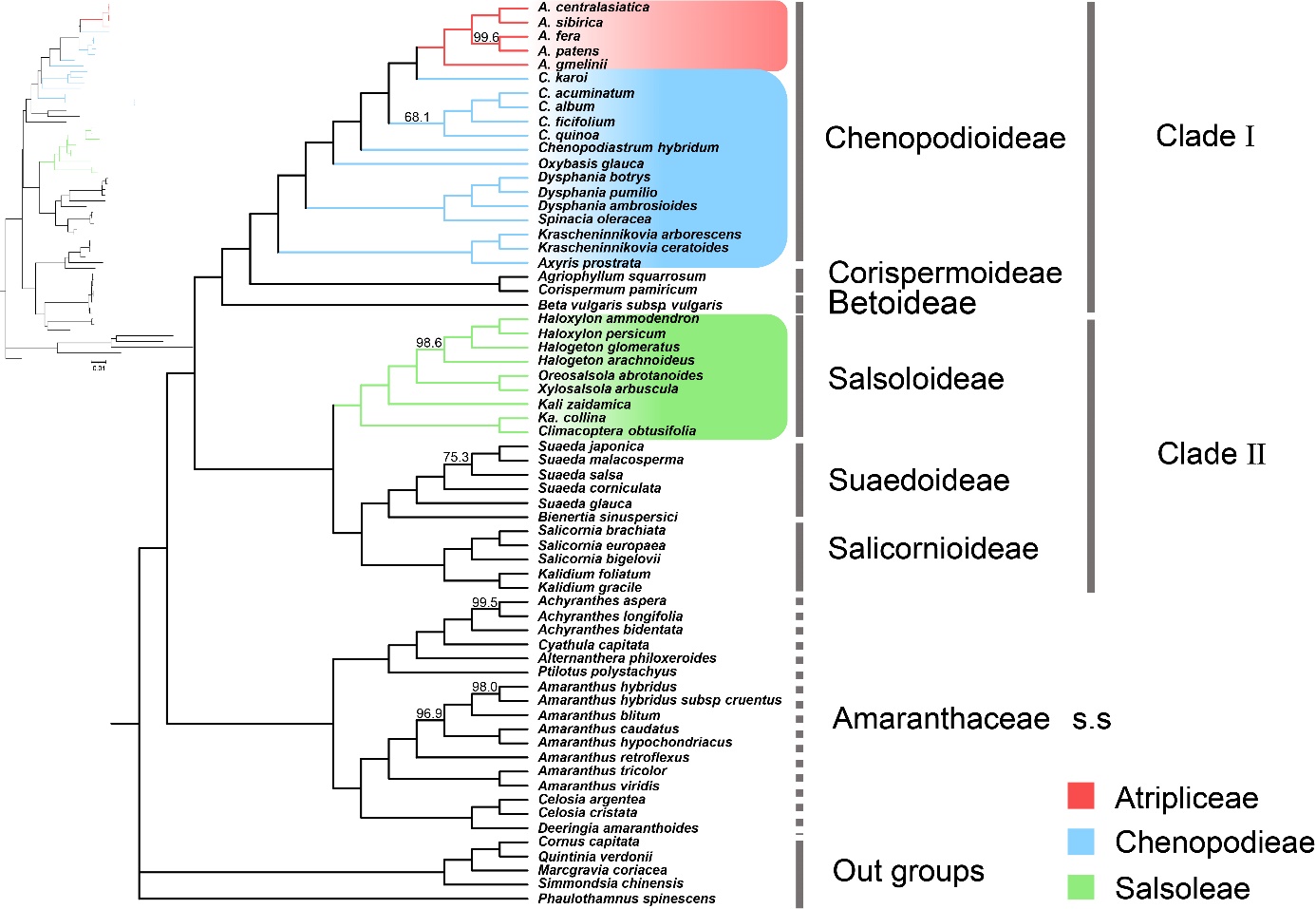


**Figure S3** Phylogenetic tree of the family Amaranthaceae *s.l.* revealed by the concatenated datasets of complete plastomes using Maximum Likelihood (ML). Support values above the branches are ML bootstrap values. Nodes without digits indicate 100% bootstrap value.


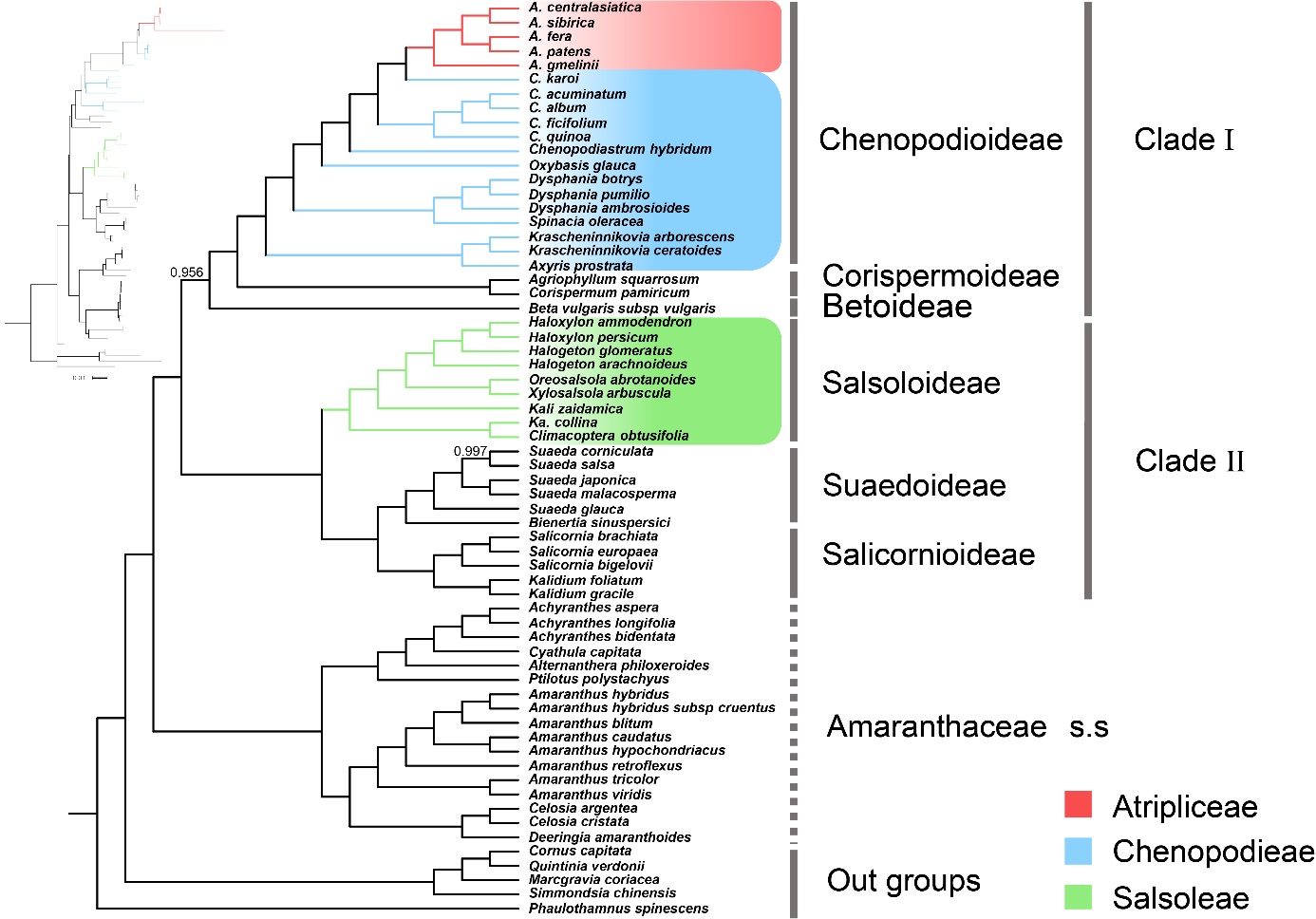


**Figure S4** Phylogenetic tree of the family Amaranthaceae *s.l.* revealed by the concatenated datasets of complete plastomes using Bayesian inference (BI). Support values above the branches are Bayesian posterior probabilities. Nodes without digits indicate 1.0 posterior probability.


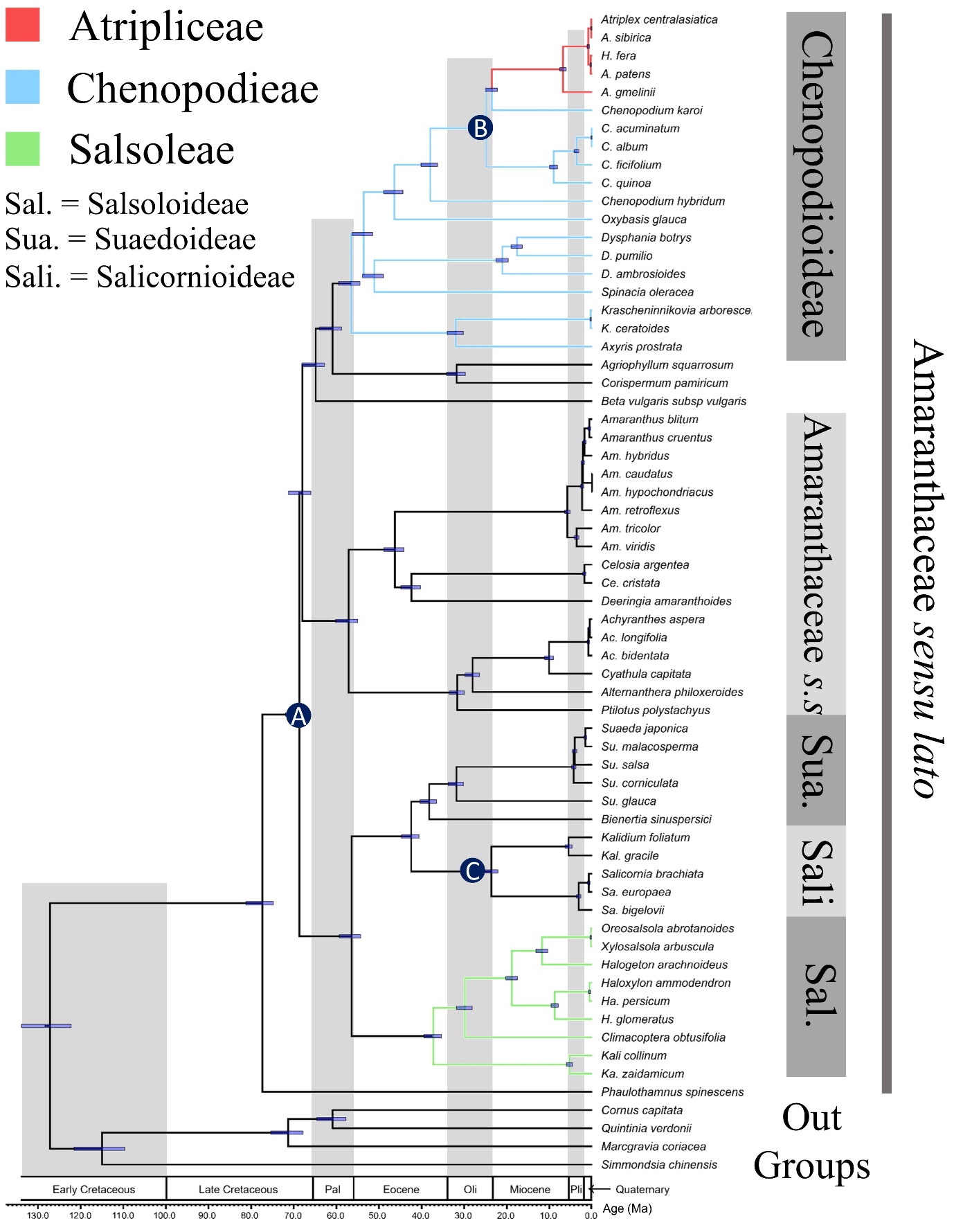


**Figure S5** Beast-derived chronogram of 59 Amaranthaceae *s.l.* using non-positively selected genes. The blue bars correspond to the 95% highest posterior density (HPD) of divergence time and the blue circles (A = *Polyporina cribraria* Srivastava, B = *Parvangula randeckensis* Hiltermann & Schmitz, C = *Salicornites massalongoi* Principi) represent three fossils to constrain divergence time.


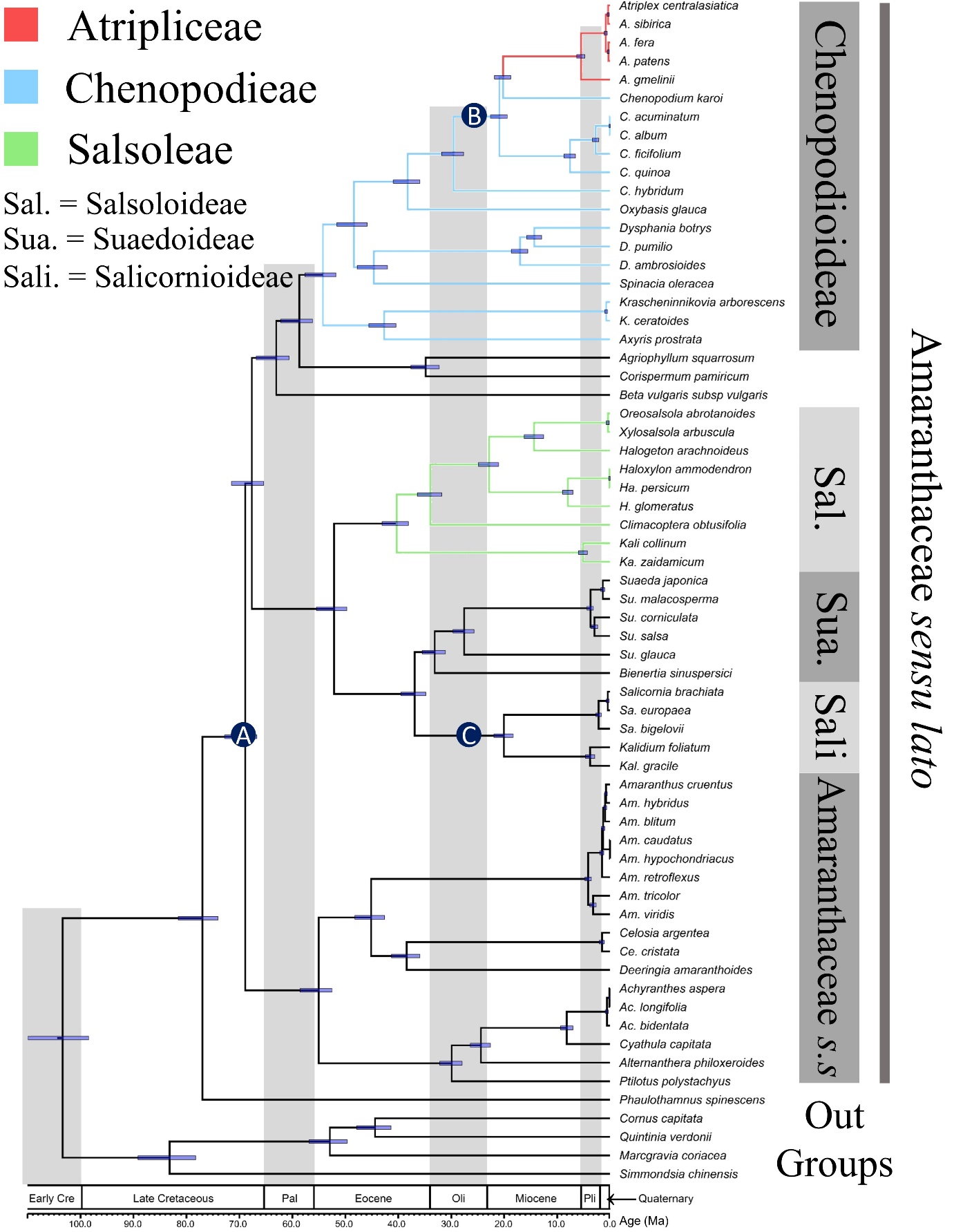


**Figure S6** Beast-derived chronogram of 59 Amaranthaceae *s.l.* using positively selected genes. The blue bars correspond to the 95% highest posterior density (HPD) of divergence time and the blue circles (A = *Polyporina cribraria* Srivastava, B = *Parvangula randeckensis* Hiltermann & Schmitz, C = *Salicornites massalongoi* Principi) represent three fossils to constrain divergence time.


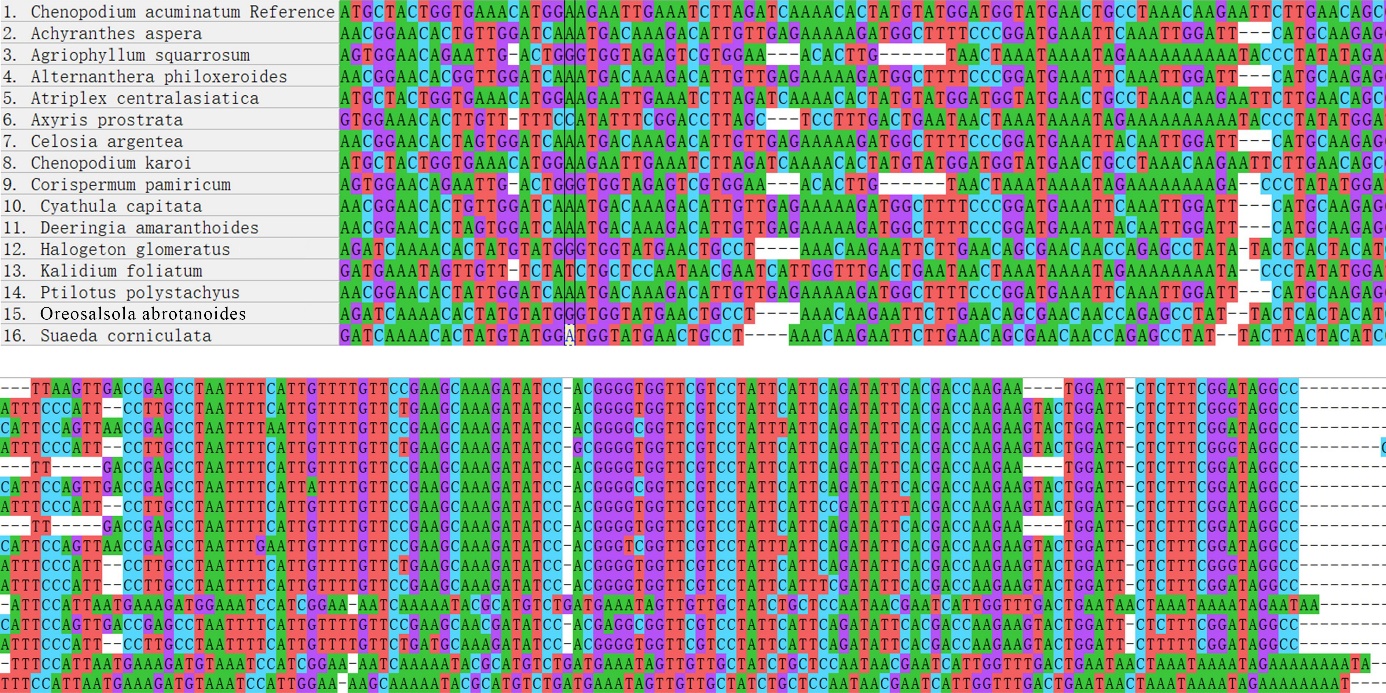


**Figure S7** Alignment of pseudo-*ycf*15 gene in fifteen representative species of Amaranthaceae *s.l.* We used *ycf*15 of *Chenopodium acuminatum* as a reference.
